# Effect of Alkyl Chain Length and Hydroxyl Substitution on the Antioxidant Activity of Gallic Acid Esters

**DOI:** 10.3390/molecules31020210

**Published:** 2026-01-07

**Authors:** Qi Chen, Shuaiwei Cui, Wenwen Zhang, Gang Dong, Baoshan Tang, Jinju Ma, Juan Xu, Jun Zhang, Lanxiang Liu

**Affiliations:** 1 Institute of Highland Forest Science, Chinese Academy of Forestry, Kunming 650233, China; chenqisjz@foxmail.com (Q.C.); cui-shuaiwei@outlook.com (S.C.); dongg1954@163.com (G.D.); tangbaos@163.com (B.T.); majinjuchem@163.com (J.M.); xujuan2006@126.com (J.X.); 2 Yunnan Key Laboratory of Breeding and Utilization of Resource Insects, Kunming 650233, China; 3 Key Laboratory of Protection and Utilization of Insects, Research Center of Engineering and Technology of Characteristic Forest Resources, National Forestry and Grassland Administration, Kunming 650233, China; 4 Hebei Technological Innovation Center for Volatile Organic Compounds Detection and Treatment in Chemical Industry, Hebei Chemical & Pharmaceutical College, Shijiazhuang 050026, China; 5 Key Laboratory of State Forestry and Grassland Administration on Highly-Efficient Utilization of Forestry Biomass Resources in Southwest China, Southwest Forestry University, Kunming 650224, China; zj8101274@163.com; 6 School of Pharmacy, Xinyang Agriculture and Forestry University, Xinyang 464000, China; zhangwenwen1105@163.com

**Keywords:** gallic acid, antioxidant, radical, alkyl esters, hydroxyl-functionalized

## Abstract

Gallic acid (GA) exhibits excellent antioxidant properties but suffers from chemical instability due to its carboxyl group, which limits practical application. To address this, we designed and investigated 14 distinct ester derivatives of GA, which were categorized into two major groups based on their substituents: chain alkyl and hydroxyl-substituted alkyl groups. Systematic evaluation revealed a striking decline in the DPPH radical scavenging activity of alkyl esters with increasing carbon chain length, from 91.9% for GA-C3 to 55.6% for GA-C30. The hydroxyl-functionalized GA esters GA-EG, GA-GL, and GA-PT maintain high antioxidant activity (>90%) while improving applicability through carboxyl substitution. In the oil system, all derivatives significantly prolong the oxidation induction time, with GA-C3 exhibiting the highest performance by extending the induction time by 2.15 h. Hydroxyl-functionalized esters such as GA-EG, GA-GL, and GA-PT also demonstrated significant efficacy, prolonging oxidation induction by 1.92 to 2.03 h. The results suggest how the structure of GA esters affects their antioxidant behavior, providing a direction for designing antioxidants suitable for specific systems.

## 1. Introduction

Gallic acid (GA) is a natural polyphenolic organic compound, which is widely present in plants such as galla chinensis, phyllanthus emblica, and rhubarb [1,2,3]. This compound exhibits a variety of significant biological activities, including antioxidant, free radical scavenging, anti-inflammatory, anti-tumor, and anti-mutagenic properties [4,5,6,7]. However, the carboxyl group in its molecular structure makes the substance sensitive to light and heat, and it is prone to decomposition, color change, and self-oxidation under high temperatures or prolonged exposure to light [8,9,10]. Additionally, its antioxidant activity shows a significant pH dependence [11,12]. Moreover, due to the extremely low solubility of GA in lipid matrices, its application in pure lipid systems such as vegetable oils, animal fats, and oil-based cosmetics is severely limited [13].

To overcome these limitations, researchers usually employ ester derivatives of GA, such as methyl gallate (GA-C1), propyl gallate (GA-C3), and octyl gallate (GA-C8) [14,15]. These ester compounds are widely used in areas such as oil antioxidants, fruit and vegetable preservation, and the synthesis of pharmaceutical intermediates [16,17]. By introducing ester groups and alkyl chains into the GA molecule, the lipid solubility is significantly improved, thereby expanding the application potential. Among them, GA-C3 is an internationally recognized food antioxidant, and its efficacy and safety are superior to synthetic antioxidants such as butylated hydroxyanisole (BHA) and butylated hydroxytoluene (BHT). After it, GA-C8 and lauryl gallate (GA-C12) also received approval from the World Health Organization and can be used as antioxidants for fried and fatty foods [18]. Studies have shown that gallate with alkyl chain carbon numbers of 3–8 exhibit excellent antioxidant performance in different oil systems, and their effects are superior to ascorbic acid [19]. In addition, Chen Xiaoxia et al. found that gallate alkyl esters can significantly alleviate lipid peroxidation damage caused by cerebral ischemia in rats [20]. Wang Zheng et al. compared the antioxidant capabilities of propyl gallate, butylate, butylate, and catechin, and found that butyl gallate performed best in in vitro experiments and had a protective effect against liver damage induced by carbon tetrachloride [21]. The above studies suggest that appropriately extending the alkyl chain length of gallate esters may help enhance their antioxidant activity and stability.

On the other hand, fatty alcohol compounds also exhibit diverse functions and applications: medium-chain fatty alcohols such as octanol (C8) and decanol (C10) have fruity aromas and are commonly used as food additives and fragrances [22,23]. Long-chain fatty alcohols such as lauryl alcohol (C12), myristyl alcohol (C14), and cetyl alcohol (C16) are important intermediates in the synthesis of surfactants and detergents [24,25,26]; while extremely long-chain fatty alcohols such as octadecanol (C18), eicosanol (C20), docosanol (C26), and triacontanol (C30) etc. [27,28,29] have been proven to have physiological effects such as reducing cholesterol, regulating blood lipids, promoting growth and development, or stimulating plant growth. In 2002, Isao Kubo et al. synthesized a series of alkyl gallate esters using GA and alcohols with different carbon chains (C6–C13) as raw materials and proved that the length of the alkyl chain has no relation to the antibacterial activity against porcine cholera *Salmonella* [30]. Wang et al. synthesized three GA esters via Steglich esterification and revealed that the presence of a branched chain or an unsaturated double bond enhances the solubility of the molecule in oil, thereby increasing its antioxidant activity [31]. However, the effects of extremely long-chain (with a carbon atom count of ≥22) GA esters, as well as the introduction of functional groups such as hydroxyl groups on the carbon chain, on the antioxidant properties of GA esters remain unexplored areas in current research.

Therefore, in this study, we synthesized a series of GA esters by reacting GA with fatty alcohols of different chain lengths. To explore the effect of functional groups, we also synthesized esters with hydroxyl groups incorporated into their side chains. We systematically evaluated the DPPH free radical scavenging ability and antioxidant activity of all target compounds in the oil system. It should be noted that among all the compounds, only GA-C26 and GA-C30 are newly synthesized (as confirmed by SciFinder). The other esters, although reported in the literature, are mostly commercially unavailable. Therefore, this paper describes the synthesis and characterization of all non-commercial compounds used in this study. This research work has laid a theoretical foundation for the development of new and highly efficient GA-based antioxidants.

## 2. Results and Discussion

### 2.1. Characterization of Alkane Chain GA Esters

**Scheme 1:**
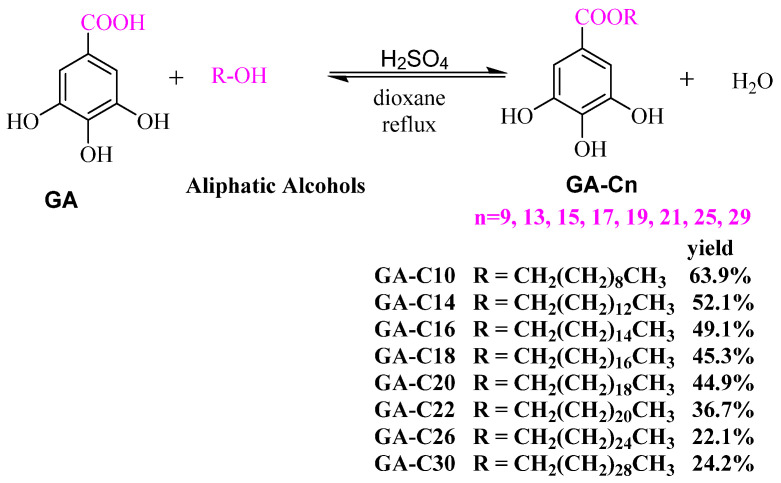
Synthesis of GA alkyl esters.

Eight GA esters with varying carbon chain lengths were synthesized (Scheme 1) via Fisher esterification reaction and their structures were confirmed by spectroscopic analysis. The results showed a significant decrease in yield as the alkyl chain lengthened, primarily due to reduced solubility of the long-chain alcohols and corresponding esters, increased steric hindrance, and challenges in subsequent isolation and extraction [32,33]. While this method has limitations in yield, its one-step simplicity and efficiency in rapidly generating a series of derivatives make it well-suited for preliminary structure-activity relationship studies. In comparison, the acyl chloride method offers higher yields but requires handling moisture-sensitive reagents [33]; enzymatic catalysis provides mild conditions and high selectivity yet at higher cost [34]; and coupling-agent-mediated approaches avoid strong acids but generate stoichiometric waste [35]. The choice of direct esterification in this work was therefore based on its practical balance between accessibility and the ability to efficiently construct a homologous series for systematic evaluation.

**Figure 1:**
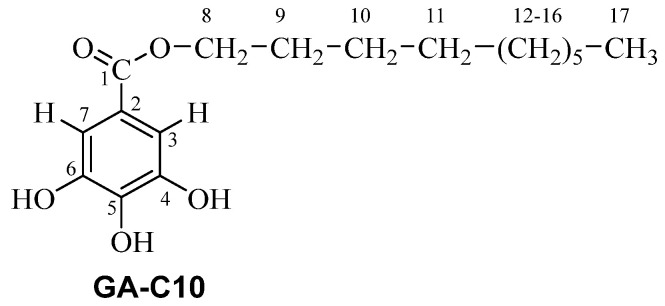
Chemical structure of GA-C10.

GA-C10 (decyl 3,4,5-trihydroxybenzoate): white solid, melting point: 92.8 °C, yield was 63.9%. IR vmax (cm^−1^): 3452, 3342, 2922, 2850, 1676, 1608, 1535, 1463, 1382, 1309, 1251, 1033, 877, 757, 640, 574. ^1^H NMR (600 MHz, CD_3_OD) δ 7.06 (s, 2H,C3, 7-H), 4.22 (t, *J* = 6.6 Hz, 2H, C8-H), 1.75–1.67 (m, 2H, C9-H), 1.47–1.41 (m, 2H, C10-H), 1.38–1.34 (m, 2H, C10-H), 1.29 (d, *J* = 6.5 Hz, 10H, C12-16-H), 0.89 (t, *J* = 7.0 Hz, 3H, C17-H). ^13^C NMR (150 MHz, CD_3_OD) δ 167.24 (C-1), 145.09 (C-3, 7), 138.31 (C-5), 120.36 (C-2), 108.63 (C-4,6), 64.41 (C-8), 31.67 (C-9), 29.31 (C-10), 29.29 (C-11), 29.06 (C-12), 29.03 (C-13), 28.50 (C-14), 25.81 (C-15), 22.34 (C-16), 13.07 (C-17). HRMS (ESI-TOF) *m*/*z* Calcd for C_17_H_26_O_5_ [M−H]^−^ 309.1780, Found 309.1713 (Figure 1).

**Figure 2:**
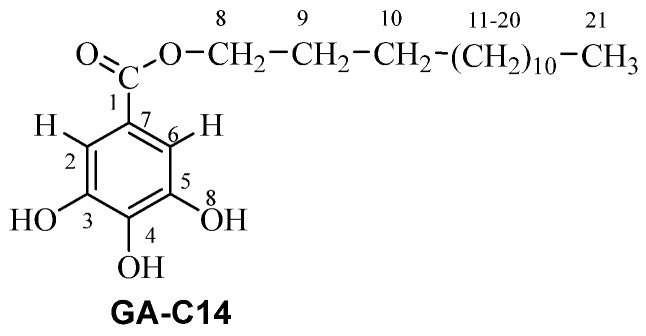
Chemical structure of GA-C14.

GA-C14 (tetradecyl 3,4,5-trihydroxybenzoate): white solid, melting point: 97.8 °C, yield was 52.1%. IR vmax (cm^−1^): 3442, 3330, 2923, 2846, 1676, 1614, 1535, 1461, 1319, 1244, 1028, 877, 756, 638. ^1^H NMR (600 MHz, DMSO-*d*_6_) δ 6.94 (s, 2H, C3, 7-H), 4.14 (t, *J* = 6.3 Hz, 2H, C8-H), 1.63 (dd, *J* = 13.7, 6.6 Hz, 2H, C9-H), 1.35 (d, *J* = 6.4 Hz, 2H, C10-H), 1.22 (s, 20H, C11, 12, 13, 14, 15, 16, 17, 18, 19, 20-H), 0.84 (t, *J* = 6.7 Hz, 3H, C21-H). ^13^C NMR (150 MHz, DMSO-*d*_6_) δ 166.32 (C-1), 146.00 (C-3, 7), 138.81 (C-5), 120.02 (C-2), 108.92 (C-4,6), 64.38 (C-8), 31.79 (C-9), 29.55 (C-10), 29.54 (C-11), 29.53 (C-12), 29.51 (C-13), 29.49 (C-14), 29.47 (C-15), 29.21 (C-16), 29.19 (C-17), 28.78 (C-18), 26.01 (C-19), 22.58 (C-20), 14.39 (C-21). HRMS (ESI-TOF) *m*/*z* Calcd for C_21_H_34_O_5_ [M−H]^−^ 365.2406, Found 365.2342 (Figure 2).

**Figure 3:**
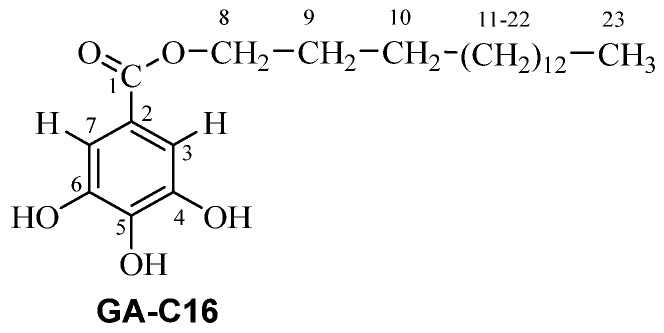
Chemical structure of GA-C16.

GA-C16 (hexadecyl 3,4,5-trihydroxybenzoate): white solid, melting point: 98.8 °C, yield was 49.1%. IR vmax (cm^−1^): 3444, 3334, 2923, 2854, 1687, 1616, 1535, 1463, 1336, 1249, 1026, 881, 759, 605, 468. ^1^H NMR (600 MHz, DMSO-*d*_6_) δ 6.94 (s, 2H, C3, 7-H), 4.14 (t, *J* = 6.4 Hz, 2H, C8-H), 1.66–1.59 (m, 2H, C9-H), 1.36 (s, 2H, C10-H), 1.22 (s, 24H, C11, 12, 13, 14, 15, 16, 17, 18, 19, 20, 21, 22-H), 0.84 (t, *J* = 6.8 Hz, 3H, C23-H). ^13^C NMR (150 MHz, DMSO-*d*_6_) δ 166.31 (C-1), 145.99 (C-3, 7), 138.80 (C-5), 120.02 (C-2), 108.92 (C-4,6), 64.37 (C-8), 31.79 (C-9), 29.55 (C-10, 11, 12, 13, 14), 29.51 (C-15, 16, 17, 18), 29.21 (C-19), 28.78 (C-20), 26.02 (C-21), 22.58 (C-22), 14.38 (C-23). HRMS (ESI-TOF) *m*/*z* Calcd for C_23_H_38_O_5_ [M−H]^−^ 393.2719, Found 393.2647 (Figure 3). 

**Figure 4:**
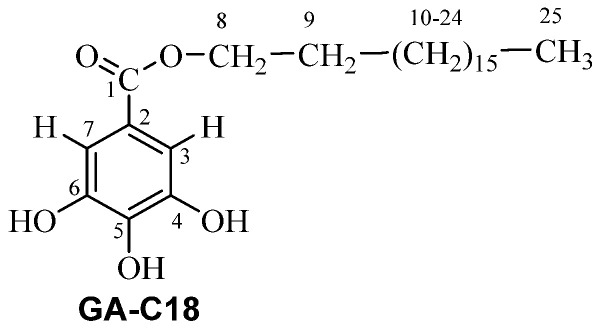
Chemical structure of GA-C18.

GA-C18 (octadecyl 3,4,5-trihydroxybenzoate): white solid, melting point: 96.6 °C, yield was 45.3%. IR vmax (cm^−1^): 3305, 2923, 2848, 1693, 1616, 1535, 1461, 1323, 1234, 1026, 879, 757, 615. ^1^H NMR (600 MHz, DMSO-*d*_6_) δ 6.94 (s, 2H, C3, 7-H), 1.60 (s, 2H, C8-H), 1.31 (s, 2H, C9-H), 1.19 (s, 30H, C10, 11, 12, 13, 14, 15, 16, 17, 18, 19, 20, 21, 22, 23, 24-H), 0.79 (d, *J* = 6.1 Hz, 3H, C25-H). HRMS (ESI-TOF) *m*/*z* Calcd for C_25_H_42_O_5_ [M−H]^−^ 421.3032, Found 421.2968 (Figure 4).

**Figure 5:**
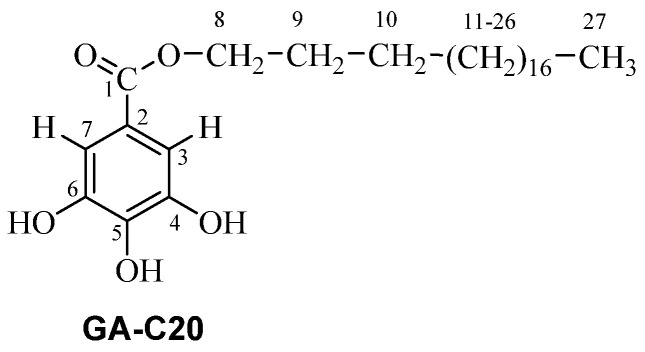
Chemical structure of GA-C20.

GA-C20 (icosyl 3,4,5-trihydroxybenzoate): white solid, melting point: 100.8 °C, yield was 44.9%. IR vmax (cm^−1^): 3319, 2910, 2844, 1689, 1614, 1535, 1463, 1319, 1190, 1028, 885, 761, 601, 474. ^1^H NMR (600 MHz, CD_3_OD) δ 7.07 (s, 2H, C3, 7-H), 4.23 (t, *J* = 6.4 Hz, 2H, C8-H), 1.74 (dd, *J* = 13.8, 6.7 Hz, 2H, C9-H), 1.47–1.43 (m, 2H, C10-H), 1.30 (s, 32H, C10, 11, 12, 13, 14, 15, 16, 17, 18, 19, 20, 21, 22, 23, 24, 25, 26-H), 0.91 (t, *J* = 6.7 Hz, 3H, C27-H). HRMS (ESI-TOF) *m*/*z* Calcd for C_27_H_46_O_5_ [M−H]^−^ 449.3345, Found 449.3280 (Figure 5).

**Figure 6:**
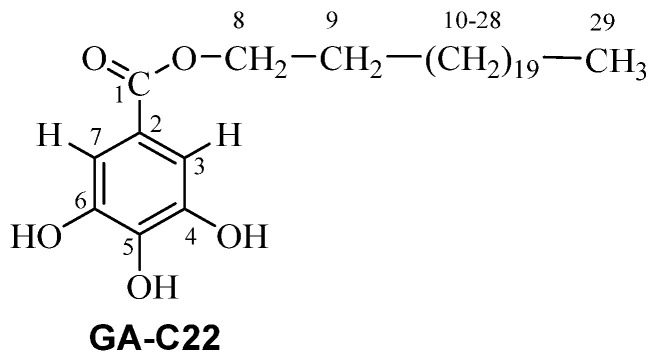
Chemical structure of GA-C22.

GA-C22 (docosyl 3,4,5-trihydroxybenzoate): white solid, melting point: 109.8 °C, yield was 36.7%. IR vmax (cm^−1^): 3332, 2914, 2848, 1703, 1608, 1529, 1463, 1338, 1199, 1037, 883, 763, 719, 638. ^1^H NMR (600 MHz, DMSO-*d*_6_) δ 6.93 (s, 2H, C3, 7-H), 1.60 (dd, *J* = 14.5, 6.9 Hz, 2H, C8-H), 1.34–1.29 (m, 2H,C9-H), 1.21 (d, *J* = 26.0 Hz, 38H, C10, 11, 12, 13, 14, 15, 16, 17, 18, 19, 20, 21, 22, 23, 24, 25, 26, 27, 28-H), 0.80 (t, *J* = 7.0 Hz, 3H, C29-H). HRMS (ESI-TOF) *m*/*z* Calcd for C_29_H_50_O_5_ [M−H]^−^ 477.3658, Found 477.3587 (Figure 6).

**Figure 7:**
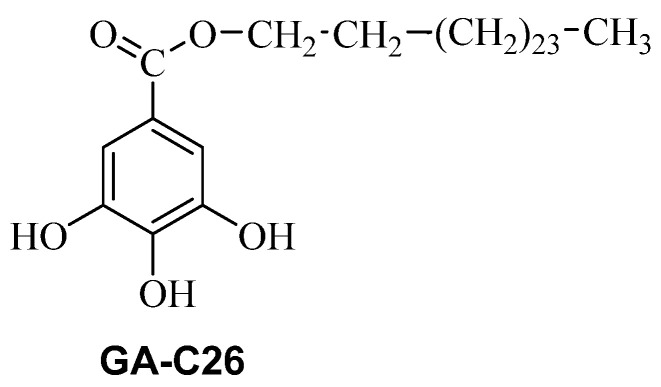
Chemical structure of GA-C26.

GA-C26 (hexacosyl 3,4,5-trihydroxybenzoate): white solid, melting point: 109.2 °C, yield was 22.1%. IR vmax (cm^−1^): 3325, 2914, 2844, 1691, 1616, 1542, 1471, 1323, 1245, 1028, 887, 763, 721, 607, 482. HRMS (ESI-TOF) *m*/*z* Calcd for C_33_H_58_O_5_ [M−H]^−^ 533.4284, Found 533.4216 (Figure 7).

**Figure 8:**
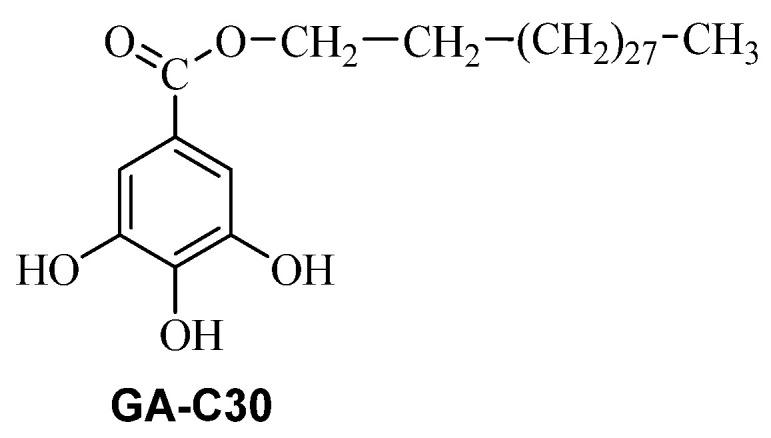
Chemical structure of GA-C30.

GA-C30 (triacontyl 3,4,5-trihydroxybenzoate): white solid, melting point: 110.7 °C, yeild was 24.2%. IR vmax (cm^−1^): 3367, 2922, 2852, 1697, 1608, 1527, 1467, 1334, 1203, 1028, 883, 767, 723, 644. HRMS (ESI-TOF) *m*/*z* Calcd for C_37_H_66_O_5_ [M−H]^−^ 589.4910, Found 589.4840 (Figure 8).

### 2.2. Characterization of GA-EG

**Scheme 2:**
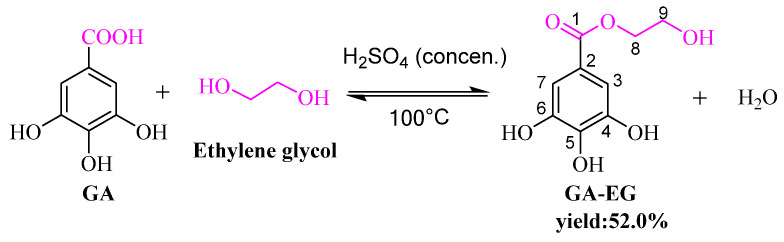
Synthesis of GA-EG.

GA-EG (2-hydroxyethyl 3,4,5-trihydroxybenzoate): white crystalline powder, melting point: 185.4 °C, yield was 52.0%. IR νmax (cm^−1^): 3379, 2920, 28546, 1687, 1606, 1537, 1454, 1400, 1325, 1240, 1024, 877, 754. ^1^H NMR (600 MHz, CD_3_OD) δ 7.07 (d, *J* = 13.5 Hz, 2H, C3, 7-H), 4.34–4.25 (m, 2H, C8-H), 3.88–3.78 (m, 2H,C9-H). HRMS (ESI-TOF) *m*/*z* Calcd for C_9_H_10_O_6_ [M−H]^−^ 213.0477, Found 213.0409 (Scheme 2).

### 2.3. Characterization of GA-GL

**Scheme 3:**
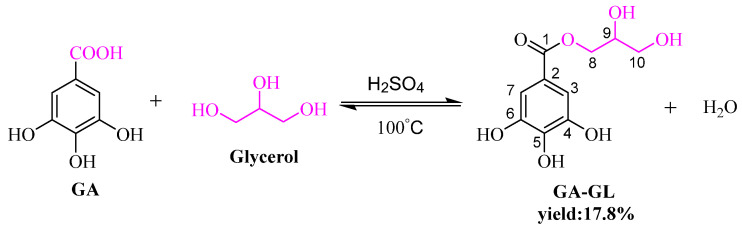
Synthesis of GA-GL.

GA-GL (2,3-dihydroxypropyl 3,4,5-trihydroxybenzoate): white crystalline powder, melting point: 243.2 °C, yield was 17.8%. IR νmax (cm^−1^): 3400, 2931, 2852, 1695, 1604, 1533, 1461, 1321, 1249, 1018, 869, 757. 1H NMR (600 MHz, CD_3_OD) δ 7.09 (s, 2H, C3, 7-H), 4.32–4.22 (m, 2H, C8-H), 3.96–3.91 (m, 1H, C9-H), 3.63 (ddt, *J* = 17.0, 11.4, 5.8 Hz, 2H, C10-H). HRMS (ESI-TOF) *m*/*z* Calcd for C_10_H_12_O_7_ [M−H]^−^ 243.0583, Found 243.0516 (Scheme 3).

### 2.4. Characterization of GA-PT

**Scheme 4:**
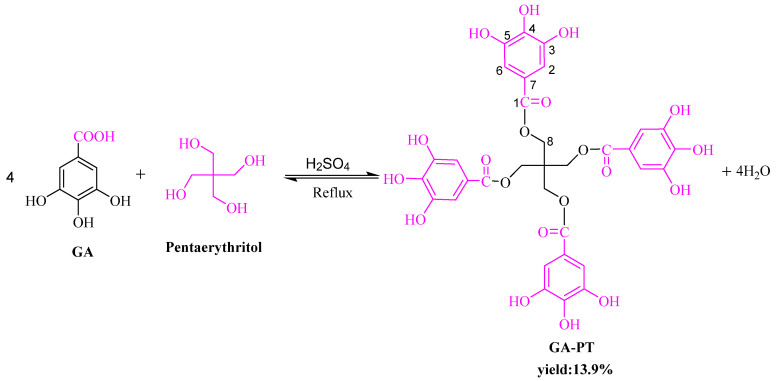
Synthesis of GA-PT.

GA-PT (2,2-bis(((3,4,5-trihydroxybenzoyl)oxy)methyl)propane-1,3-diyl bis(3,4,5-trihydroxybenzoate)): white crystalline powder, melting point: 326.3 °C, yield was 13.9%. IR νmax (cm^−1^): 3409, 1705, 1615, 1537, 1456, 1391, 1317, 1216, 1098, 1030, 864, 755, 651, 536. ^1^H NMR (600 MHz, CD_3_OD) δ 7.06 (s, 2H, C2, 6-H), 4.57–4.44 (m, 2H, C8-H). HRMS (ESI-TOF) *m*/*z* Calcd for C_33_H_28_O_20_ [M−H]^−^ 743.1174, Found 743.1100 (Scheme 4).

### 2.5. Solubility

We evaluated the solubility of GA and its five representative esters in 12 different solvents. As summarized in Table 1, GA, the short-chain ester GA-C3, and the hydroxyl-containing esters GA-EG, GA-GL, and GA-PT exhibited high solubility in hydrophilic solvents such as methanol, ethanol, DMSO, and DMF. In contrast, the extremely long alkyl chain GA-C28 was insoluble in most hydrophilic solvents, with solubility of only 3.4, 0.7, and 0.9 mg·mL^−1^ in ethanol, DMF and 1,4-dioxane, respectively. In hydrophobic solvents, neither GA nor its esters dissolved in PE or cyclohexane. However, GA and GA-C3 showed high solubility in ethyl acetate, while the other esters were only slightly soluble. In oils, the solubility of GA and GA-GL was approximately 0.4 mg·mL^−1^, followed by GA-EG and GA-PT, whereas GA-C28 was nearly insoluble but could be uniformly dispersed. These results indicate that the long alkyl chain in GA-C28 enhances its hydrophobicity and limits its solubility in nonpolar organic solvents. In contrast, the short polyhydroxy in GA-EG, GA-GL, and GA-PT impart amphiphilicity, enabling their dissolution in alcohols and medium-polarity solvents. Overall, this study provides practical guidance for solvent selection in applications involving GA esters.

### 2.6. The Effect of GA Esters in Scavenging DPPH Free Radicals

**Figure 9:**
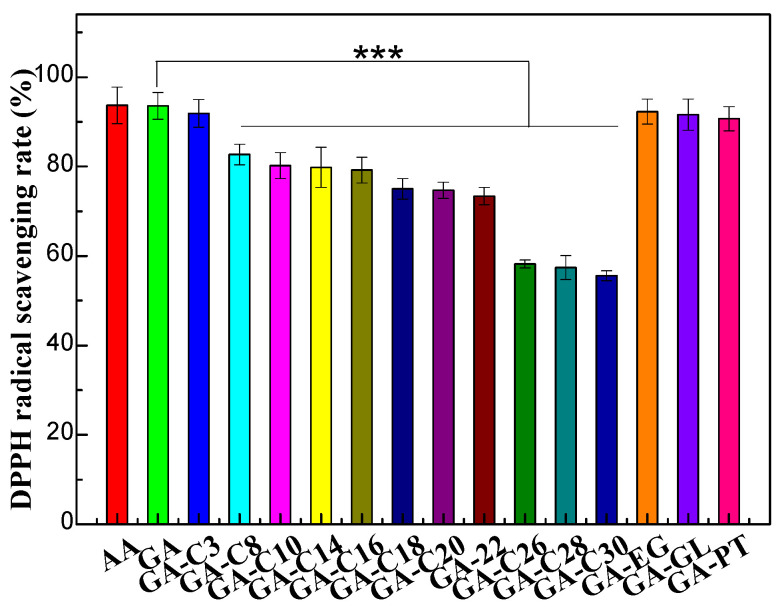
Scavenging rates of DPPH free radicals by different GA esters. Explanation: The asterisks (***) indicate statistical significance (*p* < 0.001).

The antioxidant properties of several GA esters were compared by measuring the DPPH free radical scavenging rate. The results are shown in Figure 9. There were significant differences in the DPPH free radical scavenging rates of different test samples. Among the GA alkyl esters, the scavenging rate of the short carbon chain ester GA-C3 was 91.9%, showing no significant difference from the GA and AA. The scavenging rates of the medium chain esters (GA-C8 and GA-C10) were 82.7% and 80.2%, while the average scavenging rate of the long chain and ultra-long chain esters (GA-C14 to GA-C22) was 76.4%; the esters with the longest carbon chains, GA-C26, GA-C28, and GA-C30, had scavenging rates of only 58.2%, 57.4%, and 55.6%, respectively. These results indicate that as the carbon chain length increases, the free radical scavenging ability of GA esters gradually decreases. This may be attributed to physicochemical factors. For short-chain esters GA-C3, high solubility and minimal steric hindrance in the assay medium allow the active phenolic hydroxyl groups to interact efficiently with DPPH radicals. In contrast, longer-chain esters such as GA-C26, GA-C28, and GA-C30 exhibit increased hydrophobicity, leading to potential molecular aggregation in solvents. This reduces the effective concentration of accessible phenolic groups at the reaction site. Furthermore, the larger molecular volume of long-chain esters increases steric hindrance, impeding the approach of the radicals to the active sites, and slows diffusion rates, collectively diminishing the scavenging efficiency within the assay timeframe [36,37,38].

In addition, the clearance rates of GA-EG, GA-GL, and GA-PT were 92.3%, 91.6%, and 90.7%, respectively, showing no significant difference from GA and AA. Moreover, these esters retained their antioxidant activity while having their carboxyl groups replaced, thereby expanding their applicable conditions and usage scope.

It is noteworthy that this comparative study primarily aimed at elucidating the structure–activity relationship (SAR) and the general trend of antioxidant potency across the GA ester series. Therefore, the DPPH assay was conducted at a single, high concentration to effectively rank and compare the relative scavenging capacities under standardized conditions. While this approach reveals the significant impact of alkyl chain length on activity, it does not provide full dose–response curves or precise IC_50_ values, which would be required for a detailed kinetic or potency quantification. Future studies focusing on the most promising candidates identified here will incorporate comprehensive concentration-dependent analyses to determine their exact IC_50_ values and elucidate the underlying reaction kinetics.

### 2.7. Evaluation of GA Esters in Oil Oxidation Prevention

**Figure 10:**
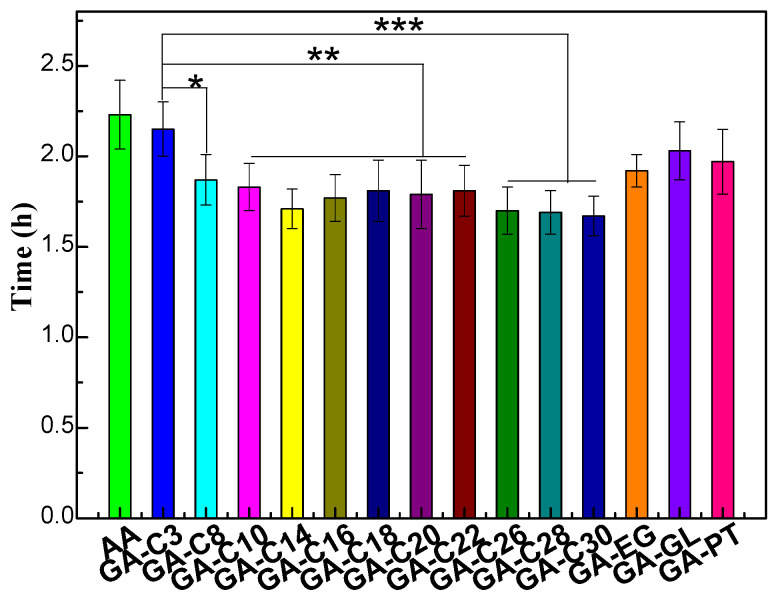
Effect of GA esters in oil oxidation prevention. Explanation: Asterisks denote statistical significance compared to the control group: * *p* < 0.05, ** *p* < 0.01, *** *p* < 0.001.

According to the method for determining the antioxidant properties of oils, the antioxidant performance of several GA esters in the oil system was evaluated. The results are shown in Figure 10. Compared with the blank control group, adding various GA esters can effectively prolong the oxidation induction time of the oil. This is mainly attributed to the phenolic hydroxyl groups in their molecules that can provide hydrogen atoms and interrupt the free radical chain reaction of lipid oxidation [39,40]. Among them, GA-C3 has the most significant antioxidant effect, extending the antioxidant time of the oil by 2.15 h, which is comparable to the extension by AA (2.23 h). This effect is closely related to its smaller molecular weight and higher phenolic hydroxyl density. In contrast, the significantly lower activity of longer-chain esters (GA-C10~GA-C22) and the extremely significant reduction for the ultra-long-chain analogs (GA-C26~GA-C30) stem from the increasing molecular bulk and declining hydroxyl density. This results in greater steric shielding of the active phenolic groups and reduced mobility within the lipid matrix, thereby diminishing their ability to efficiently scavenge radicals and impart antioxidant protection [36,37,40]. In addition, the GA esters containing hydroxyl groups, such as GA-EG, GA-GL, and GA-PT, which prolong the oxidation time by 1.92 h, 2.03 h, and 1.97 h, respectively, also exhibit good antioxidant activity, and their effects are not significantly different from GA-C3 and each other. These derivatives enhance the balance of lipophilicity and hydrophilicity of the molecule by introducing additional hydroxyl groups, enabling it to disperse well in the oil phase while maintaining its effective free radical scavenging ability [41,42,43]. The results show that the GA esters examined all have the potential to be used as oil antioxidants, and their antioxidant efficacy mainly depends on the activity of the phenolic hydroxyl groups and the spatial distribution state of the molecules in the oil. In practical applications, they can be flexibly selected according to specific usage scenarios.

## 3. Materials and Methods

### 3.1. Materials

Monohydrated GA, GA propyl ester (GA-C3), GA dodecyl ester (GA-C12), all alcohols (with purity of 95% for C12–C20 and 90% for C22-C30), ethylene glycol, propylene glycol, pentaerythritol, ascorbic acid (AA), and 1,1-diphenyl-2-picrylhydrazyl (DPPH) were purchased from Aladdin Biochemical Technology Co., Ltd. (Shaihai, China)., while organic solvents such as *N*,*N*-Dimethylformamide (DMF), Dimethyl sulfoxide (DMSO), and Petroleum ether (PE) were purchased from Guangdong Guanghua Technology Co., Ltd. (Shantou, China).

### 3.2. Synthesis of GA Alkyl Esters

Monohydrated GA (3.0 equiv., 12.0 mmol, 2.26 g) was placed in a double-mouth round-bottomed flask. Then, 4.0 mmol (1.0 equiv.) of aliphatic alcohols (C10, C14, C16, C18, C20, C22, C26, C30,) and 20.0 mL of 1,4-dioxane was added sequentially, followed by the addition of concentrated sulfuric acid of the same mass as the alkanol. The mixture was heated under reflux for 24 h. After the reaction mixture was cooled, the 1,4-dioxane was first removed under reduced pressure at a temperature not exceeding 60 °C to obtain a concentrated residue. This residue was then taken up in 50 mL ice water. The aqueous layer was separated and extracted with ethyl acetate (3 × 70 mL). The combined organic layers were washed with 30 mL NaHCO_3_, dried over anhydrous Na_2_SO_4_, and filtered. Then, the solvent was removed by reduced-pressure distillation to afford the crude product. The crude product was purified by column chromatography silica gel (stationary phase: SilicaFlash^®^ F60, 40–63 μm silica gel (Sigma-Aldrich, Shanghai, China); column dimensions: 4.0 cm diameter × 40 cm height), with the elution system being ethyl acetate/petroleum ether/formic acid (*v*/*v*/*v* = 6:4:0.03). Additionally, GA-C28 was synthesized according to our previously reported procedure [29].

### 3.3. Synthesis of GA-EG

In a single-necked round-bottomed flask equipped with a water separator, combine monohydrated GA (1.0 equiv., 20.0 mmol, 3.76 g), ethylene glycol (20.0 equiv., 22.3 mL, 400 mmol), cyclohexane (10.0 mL), and concentrated sulfuric acid (0.19 equiv., 0.2 mL, 3.8 mmol) as a catalyst. The reaction was carried out at 100 °C for 48 h. Upon completion of the reaction, the mixture was diluted with 100.0 mL of deionized water and extracted with ethyl acetate (3 × 100.0 mL). The combined organic phases were dried over anhydrous sodium sulfate, filtered, and concentrated under reduced pressure to afford the crude product. Purify the resulting crude product by column chromatography (eluent: ethyl acetate/petroleum ether/formic acid, 6:4:0.06, *v*/*v*/*v*).

### 3.4. Synthesis of GA-GL

Monohydrated GA (1.0 equiv., 20.0 mmol, 3.76 g), glycerol (20.0 equiv., 400.0 mmol, 29.2 mL), cyclohexane (10.0 mL), and concentrated sulfuric acid (0.19 equiv., 0.2 mL, 3.8 mmol) as the catalyst were added to a single-necked flask equipped with a Dean–Stark apparatus. The reaction was carried out at 100 °C for 48 h. Upon completion, the product was subsequently worked up and purified according to the procedure described in Section 3.3.

### 3.5. Synthesis of GA-PT

Monohydrated GA (4.1 equiv., 20.5 mmol, 3.86 g), pentaerythritol (1.0 equiv., 5.0 mmol, 0.68 g), 1,4-dioxane (20.0 mL), and cyclohexane (10.0 mL) were added to a single-necked, round-bottomed flask equipped with a Dean–Stark apparatus. Concentrated sulfuric acid (4.0 equiv., 1.1 mL, 20 mmol) was then introduced. The reaction mixture was heated to reflux with stirring for 24 h. The product was subsequently worked up and purified according to the procedure described in Section 3.3.

### 3.6. Characterization

All synthesized GA esters are mono-substituted at the carboxyl group, with the three phenolic hydroxyl groups remaining free. This was a deliberate design to preserve the antioxidant pharmacophore.

The organic functional groups of the samples were characterized by Fourier transform infrared (FTIR) spectroscopy using a Tenson 27 spectrometer (Bruker, Billerica, MA, USA). Spectra were recorded in the range of 4000–400 cm^−1^ with a resolution of 4 cm^−1^ to identify characteristic absorption peaks.

Additionally, nuclear magnetic resonance (NMR) spectra were acquired on a Bruker Avance 600 spectrometer (Bruker BioSpin AG, Fällanden, Switzerland) using DMSO-*d*_6_ or CD_3_OD as the solvents. Chemical shifts (δ) are reported in parts per million (ppm) relative to tetramethylsilane (TMS) as an internal reference. Purity was confirmed to be >95% by ^1^H NMR analysis, with no detectable signals corresponding to unreacted gallic acid or other impurities. High-resolution mass spectrometry (HRMS) data were acquired in negative ESI mode on an Agilent 6540 Q-TOF LC/MS system (Agilent Technologies, Santa Clara, CA, USA).

### 3.7. Determination of the Solubility

Accurately weighed 5.0 mg of the sample was placed in a stoppered test tube, followed by the addition of 5.0 mL of a specific solvent. The mixture was oscillated for 5 min at room temperature and then sonicated for 30 min to ensure complete dissolution and equilibrium. If the sample dissolved completely, additional quantified aliquots were successively added until saturation was reached. The saturated solution was subsequently kept at room temperature for 2 h, after which the undissolved solid was filtered, dried, and weighed. The solubility (*S*, mg·mL^−1^) was calculated according to Formula (1):

*S* = (m_0_ − m_1_)/5 (1)

where m_0_ and m_1_ are the total mass of the sample added and the mass of the undissolved solid (mg), respectively.

### 3.8. Determination of DPPH Radical Scavenging Capacity

Accurately weigh each GA ester sample, using anhydrous ethanol as the solvent, to prepare a sample stock solution with a concentration of 0.5 mmol·L^−1^. Simultaneously, prepare a DPPH-anhydrous ethanol working solution with a concentration of 0.2 mmol·L^−1^.

Take 0.5 mL of the sample solution and mix it with 7.0 mL of the DPPH working solution. Place it at room temperature in the dark for 1 h, then measure its absorbance at 517 nm using an enzyme reader and record it as A_1_. Set up the sample local control (A_2_): 0.5 mL of the sample solution mixed with 7.0 mL of anhydrous ethanol. The control group containing only the DPPH solution (A_0_): 0.5 mL of anhydrous ethanol mixed with 7.0 mL of the DPPH working solution. Also, use 0.5 mmol·L^−1^ AA as the positive control. The DPPH radical scavenging rate is calculated according to the following Formula (2):

DPPH radical scavenging rate (%) = [(A_0_ − (A_1_ − A_2_))/A_0_] × 100% (2)

### 3.9. Oxidation Resistance Measurement of Oil

The antioxidant performance of the samples is evaluated by determining their induction period under accelerated oxidation conditions. The experiment is conducted in the oil oxidation instrument (VELP Scientifica Srl, Usmate Velate, Italy). The specific steps are as follows: Using refined rapeseed oil as the base, a series of oil samples containing different antioxidants are precisely prepared. The concentration of all additives is 0.4 mmol·L^−1^. The control is plain rapeseed oil. After the samples are prepared, the above oil samples are accurately weighed 10.00 ± 0.01 g into the dedicated sample trays. The sample tray is placed in the oxidation instrument, and accelerated oxidation is carried out at a constant temperature of 110 °C and an oxygen pressure of 6 Pa. For each test, two parallel reaction chambers are set up: Chamber A is fixed with the blank control oil sample, and Chamber B is the sample to be tested. The relative efficacy of each antioxidant is evaluated by comparing the oxidation induction time of the samples with that of the blank control. Also, use 0.4 mmol·L^−1^ AA as the positive control.

### 3.10. Statistical Analysis

Data from three independent experiments are expressed as mean ± SD. Statistical significance was assessed by one-way ANOVA test (* *p* < 0.05, ** *p* < 0.01, *** *p* < 0.001).

## 4. Conclusions

This study designed and investigated two major comprising 14 GA ester derivatives and systematically evaluated their antioxidant activities. The results demonstrated that the antioxidant efficacy is governed by the number of phenolic hydroxyl groups and their distribution in the reaction medium. In the DPPH assay, alkyl gallates showed significant chain-length dependence. The short-chain ester GA-C3 exhibited the highest scavenging rate of 91.9%, while the long-chain esters GA-C26 and GA-C30 showed significantly reduced activities of 58.2% and 55.6%, respectively. This decrease is attributed to the molecular aggregation and increased steric hindrance. In contrast, the hydroxyl-functionalized esters GA-EG, GA-GL, and GA-PT maintained high scavenging activities of 90.7% to 92.3%, comparable to pristine GA, indicating that carboxyl group substitution does not impair their radical-scavenging capacity. In the lipid oxidation system, all derivatives effectively prolonged the oxidation induction time. GA-C3 showed the best performance with an extension of 2.15 h, while the hydroxyl-functionalized esters also demonstrated strong efficacy, extending the induction time by 1.92 to 2.03 h due to their good dispersibility and retained activity in the oil phase. This work provides a theoretical foundation for the rational design of GA ester antioxidants tailored for specific applications. Future studies will build upon this foundation by scaling up synthesis to enable full dose-response characterization and precise IC_50_ determination for tailored applications.

## Figures and Tables

**Table 1:** Solubility of representative compounds in different solvents.

		GA	PG	GA-C28	GA-EG	GA-GL	GA-PT
Entrey Solvents					Solubility (mg·mL^−1^)		
1	Water	≈16.6	≈7.5	0	≥50.0	≥50.0	≥50.0
2	methanol	≥50.0	≥50.0	0	≥50.0	≥50.0	≥50.0
3	Ethanol	≥50.0	≥50.0	≈3.4	≥50.0	≥50.0	≥50.0
4	DMSO	≥50.0	≥50.0	0	≥50.0	≥50.0	≥50.0
5	DMF	≥50.0	≥50.0	≈0.7	≥50.0	≥50.0	≥50.0
6	1,4-Dioxane	≥50.0	≥50.0	≈0.9	≥50.0	≥50.0	≥50.0
7	Acetone	≥50.0	≥50.0	≈0.2	≈0.2	≈0.6	≈0.1
8	Glycerol	≥50.0	≥50.0	0	≈2.8	≈4.3	≈1.1
9	PE	0	0	0	0	0	0
10	Cyclohexane	0	0	0	0	0	0
11	Ethyl acetate	≥50.0	≥50.0	≈2.3	≈3.5	≈2.0	≈1.2
12	oil	≈0.4	≈0.1	0	≈0.2	≈0.4	≈0.2

## Data Availability

The original contributions presented in this study are included in the article and Supplementary Materials. Further inquiries can be directed to the corresponding author.

## Supplementary Materials

The following supporting information can be downloaded at: https://www.mdpi.com/article/10.3390/molecules31020210/s1, the supplementary information includes the NMR spectra, along with the low- and high-resolution mass spectra, for all synthesized compounds reported in the main text.
