# Supplementary material for: Effect of Alkyl Chain Length and Hydroxyl Substitution on the Antioxidant Activity of Gallic Acid Esters
Source: Molecules. 2026 Jan 7;31(2):210. doi: 10.3390/molecules31020210 (PMC12844035; doi:10.3390/molecules31020210)
Supplement: Supplementary file 1 [file molecules-31-00210-s001.zip › molecules-4063218-supplementary.pdf]

Effect of Alkyl Chain Length and Hydroxyl Substitution on the Antioxidant Activity of Gallic Acid Esters

Qi Chen [1,2], Shuaiwei Cui [1,3], Wenwen Zhang [4], Gang Dong [1], Baoshan Tang [1], Jinju Ma [1], Juan Xu [1], Jun Zhang [3] and Lanxiang Liu [1,*]

[1] Institute of Highland Forest Science, Chinese Academy of Forestry. Yunnan Key Laboratory of Breeding and Utilization of Resource Insects. Key Laboratory of Protection and Utilization of Insects, Research Center of Engineering and Technology of Characteristic Forest Resources, National Forestry and Grassland Administration, Kunming 650233, China; chenqisjz@foxmail.com (Q.C.); cui-shuaiwei@outlook.com (S.C.); dongg1954@163.com (G.D.); tangbaos@163.com (B.T.); majinjuchem@163.com (J.M.); xujuan2006@126.com (J.X.)

[2] Hebei Technological Innovation Center for Volatile Organic Compounds Detection and Treatment in Chemical Industry, Hebei Chemical & Pharmaceutical College, Shijiazhuang 050026, China

[3] Key Laboratory of State Forestry and Grassland Administration on Highly-Efficient Utilization of Forestry Biomass Resources in Southwest China, Southwest Forestry University, Kunming 650224, China; zj8101274@163.com

[4] School of Pharmacy, Xinyang Agriculture and Forestry University, Xinyang 464000, China; zhangwenwen1105@163.com

* Correspondence: lanxiangliu@outlook.com

CONTENT

Figure S1. 1H NMR spectroscopy of GA-C10
Figure S2. 13C NMR and DEPT spectroscopy of GA-C10
Figure S3. Mass spectrum of GA-C10
Figure S4. High resolution mass spectrum of GA-C10
Figure S5. 1H NMR spectroscopy of GA-C14
Figure S6. 13C NMR and DEPT spectroscopy of GA-C14
Figure S7. Mass spectrum of GA-C14
Figure S8. High resolution mass spectrum of GA-C14
Figure S9. 1H NMR spectroscopy of GA-C16
Figure S10. 13C NMR and DEPT spectroscopy of GA-C16
Figure S11. Mass spectrometr of GA-C16
Figure S12. High resolution mass spectrum of GA-C16
Figure S13 1H NMR spectroscopy of GA-C18
Figure S14. Mass spectrum of GA-C18
Figure S15. High resolution mass spectrum of GA-C18
Figure S16. 1H NMR spectroscopy of GA-C20
Figure S17. Mass spectrum of GA-C20
Figure S18. High resolution mass spectrum of GA-C20
Figure S19. 1H NMR spectroscopy of GA-C22
Figure S20. Mass spectrum of GA-C22
Figure S21. High resolution mass spectrum of GA-C22
Figure S22. Mass spectrum of GA-C26
Figure S23. High resolution mass spectrum of GA-C26
Figure S24. Mass spectrum of GA-C30
Figure S25. High resolution mass spectrum of GA-C30
Figure S26. 1H NMR spectroscopy of GA-EG
Figure S27. Mass spectrum of GA-EG
Figure S28. High resolution mass spectrum of GA-EG
Figure S29. 1H NMR spectroscopy of GA-GL
Figure S30. Mass spectrum of GA-GL
Figure S31. High resolution mass spectrum of GA-GL
Figure S32. 1H NMR spectroscopy of GA-PT
Figure S33. Mass spectrum of GA-PT
Figure S34. High resolution mass spectrum of GA-PT

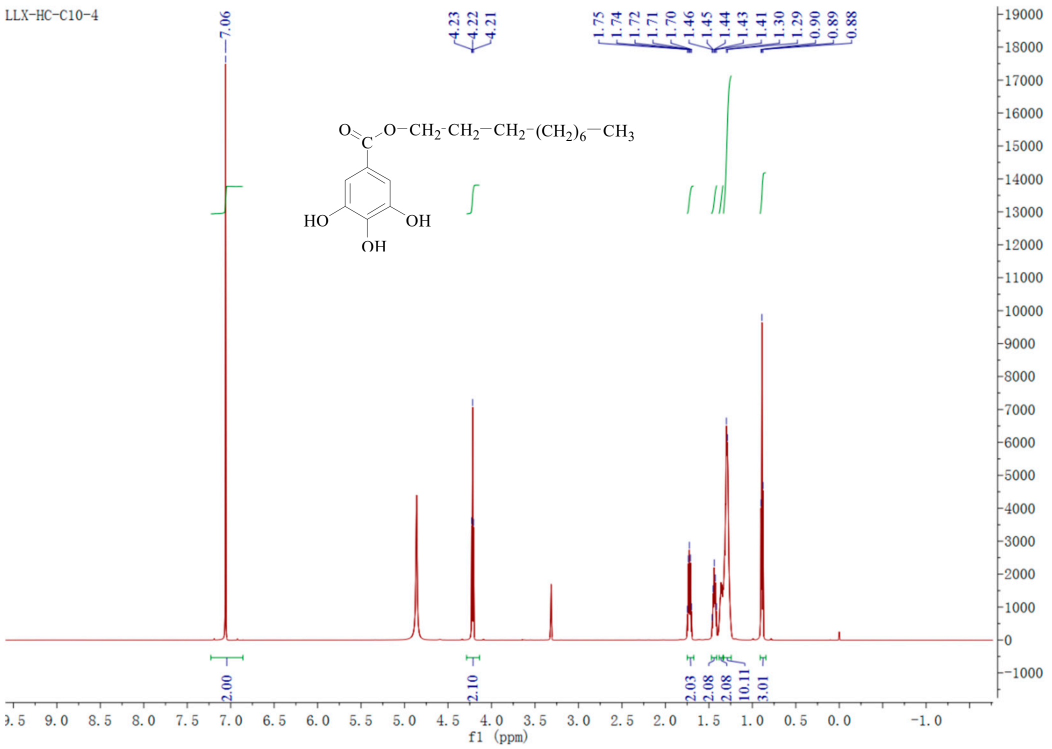

Figure S1. ^{1}H NMR spectroscopy of GA-C10

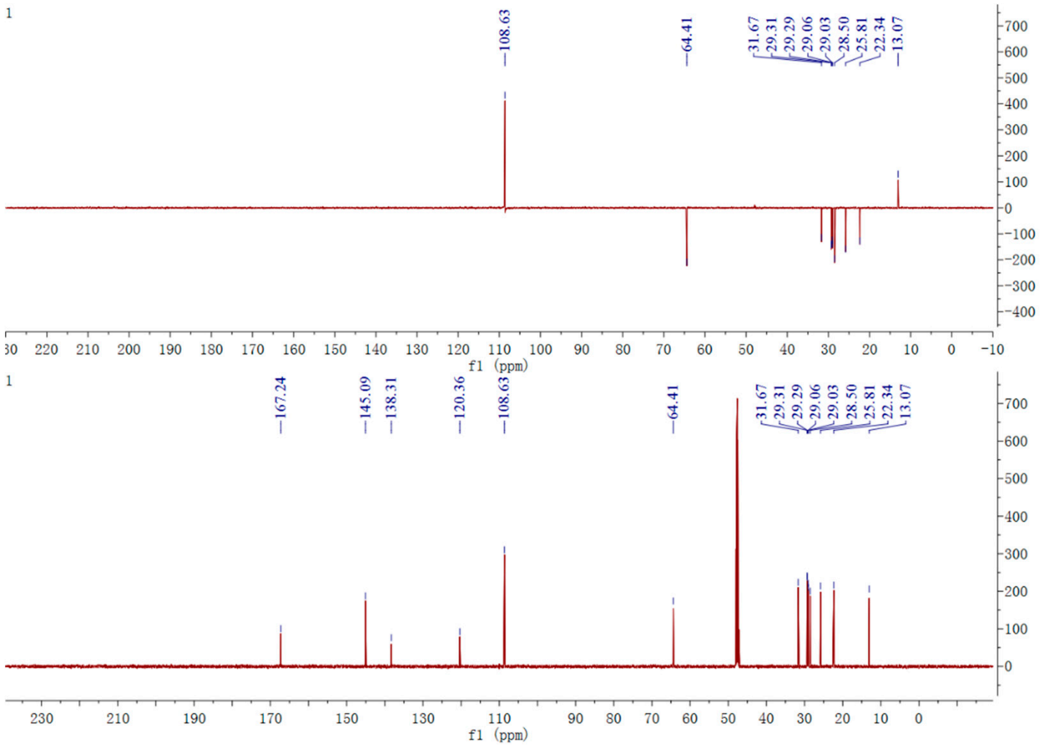

Figure S2. ^{13}C NMR and DEPT spectroscopy of GA-C10

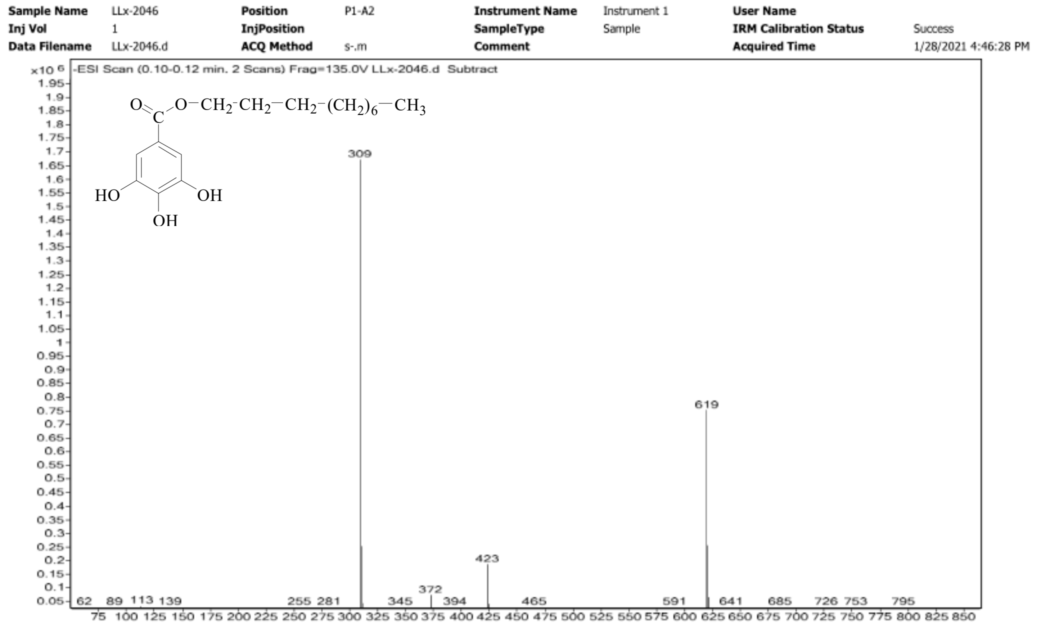

Figure S3. Mass spectrum of GA-C10

Mass spectrometric conditions:

Mass spectrometric studies were carried out on a quadrupole time-of-flight (Q-TOF) high-resolution mass spectrometer (Q-TOF LC/MS 6540 series, Agilent Technologies, Santa Clara, CA, USA) coupled with electrospray ionization (ESI). The data was acquired using Mass Hunter Workstation software. The detection was performed in negative ESI mode. The MS parameters were optimized as follows: the fragmentor voltage was set at 135 V; the capillary was set at 3500 V; the skimmer was set at 65 V; and nitrogen was used as the drying (350 °C, 9 L/min) and nebulizing (40 psi) gas.

Qualitative Analysis Report

**Data Filename**	LLx-2046.d	**Sample Name**	LLx-2046
**Sample Type**	Sample	**Position**	P1-A2
**Instrument Name**	Instrument 1	**User Name**	
**Acq Method**	s-.m	**Acquired Time**	1/28/2021 4:46:28 PM
**IRM Calibration Status**	Success	**DA Method**	PCDL.m
**Comment**			

Sample Group **Info.**

Acquisition SW Version 6200 series TOF/6500 series Q-TOF B.05.01 (B5125.2)

User Spectra

Fragmentor Voltage	Collision Energy	Ionization Mode
135	0	ESI

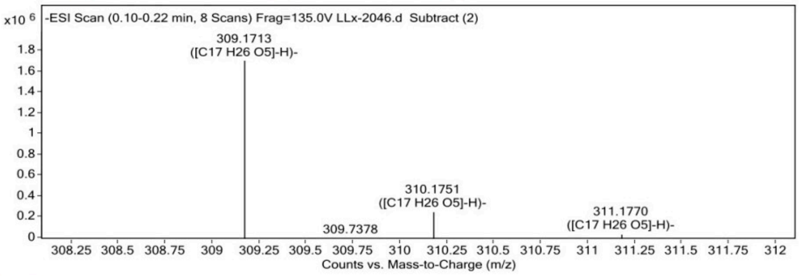

Peak List

m/z	z	Abund	Formula	Ion
309.1713	1	1703101.38	C17 H26 O5	(M-H)-
310.1751	1	255726.77	C17 H26 O5	(M-H)-
311.177	1	43225.71	C17 H26 O5	(M-H)-
372.1668	1	71930.03		
423.1646	1	182663.28		
424.1674	1	39683.54		
619.3499	1	812906.75		
620.3537	1	268415.28		
621.356	1	67749.08		
1230.1732	1	40121.16		

Formula Calculator Element Limits

Element	Min	Max
C	3	50
H	0	100
O	0	20

Formula Calculator Results

Formula	CalculatedMass	CalculatedMz	Mz	Diff. (mDa)	Diff. (ppm)	DBE
C17 H26 O5	310.1780	309.1707	309.1713	-0.60	-1.94	5.0000

--- End Of Report ---

Figure S4. High resolution mass spectrum of GA-C10

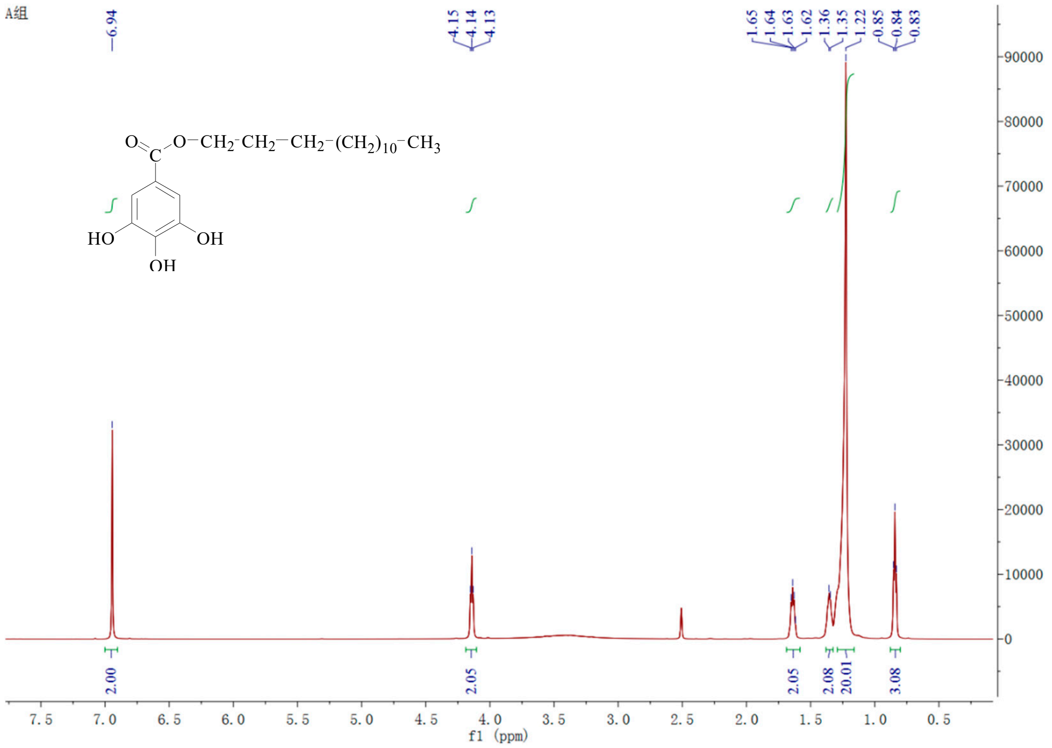

Figure S5. ^{1}H NMR spectroscopy of GA-C14

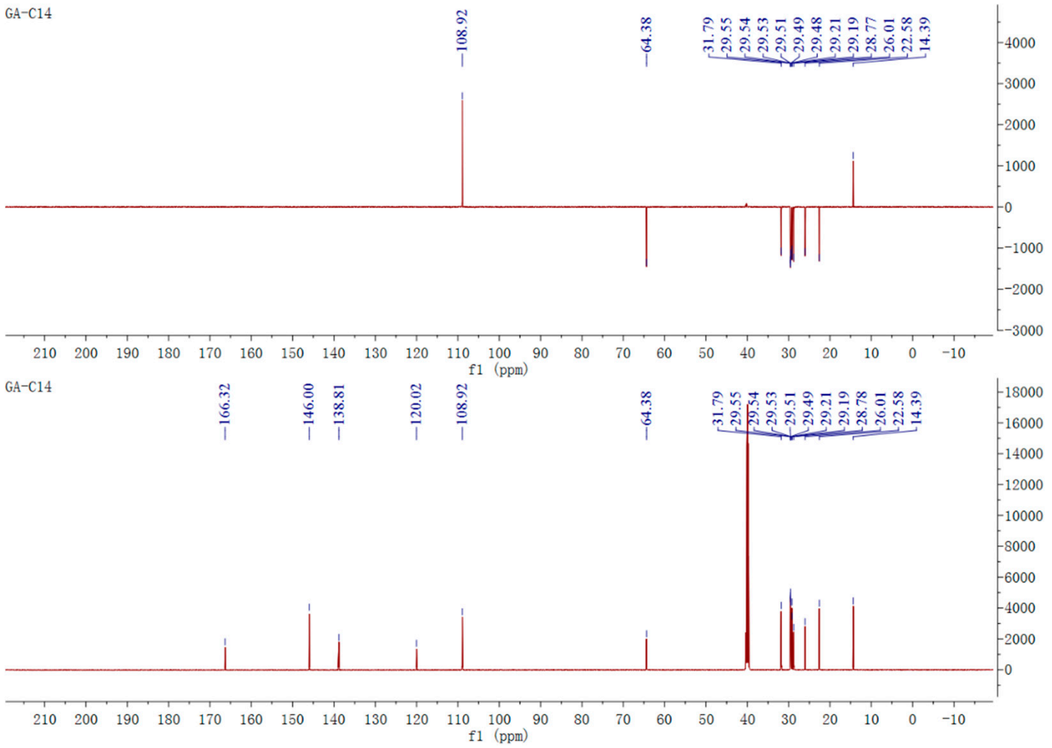

Figure S6. ^{13}C NMR and DEPT spectroscopy of GA-C14

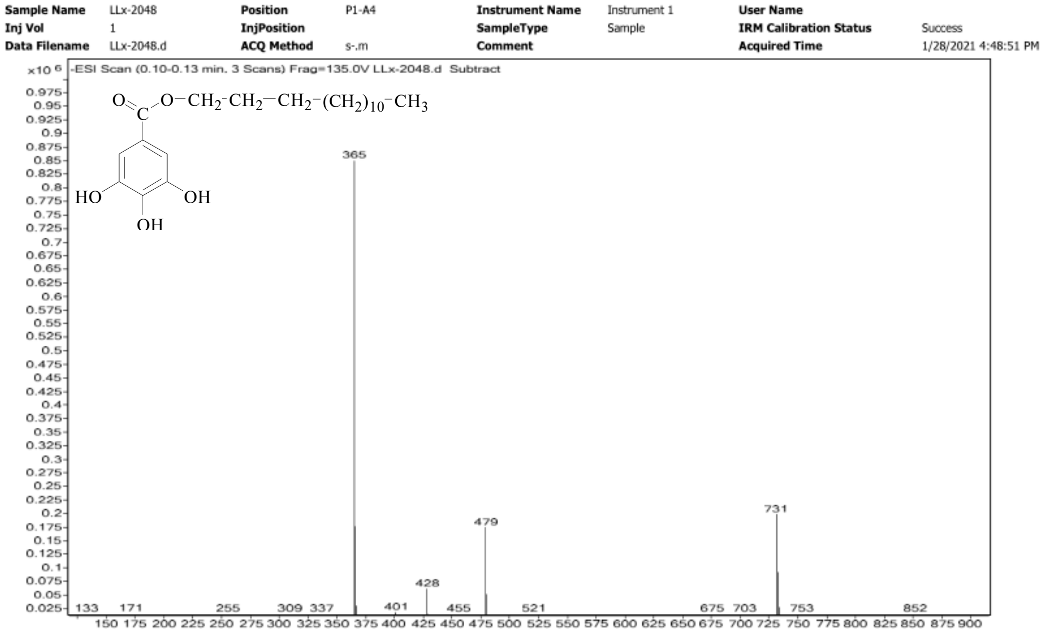

Figure S7. Mass spectrum of GA-C14

Qualitative Analysis Report

**Data Filename**	LLx-2048.d	**Sample Name**	LLx-2048
**Sample Type**	Sample	**Position**	P1-A4
**Instrument Name**	Instrument 1	**User Name**	
**Acq Method**	s-.m	**Acquired Time**	1/28/2021 4:48:51 PM
**IRM Calibration Status**	Success	**DA Method**	PCDL.m
**Comment**			

Sample Group **Info.**

Acquisition SW Version 6200 series TOF/6500 series Q-TOF B.05.01 (B5125.2)

User Spectra

Fragmentor Voltage	Collision Energy	Ionization Mode
135	0	ESI

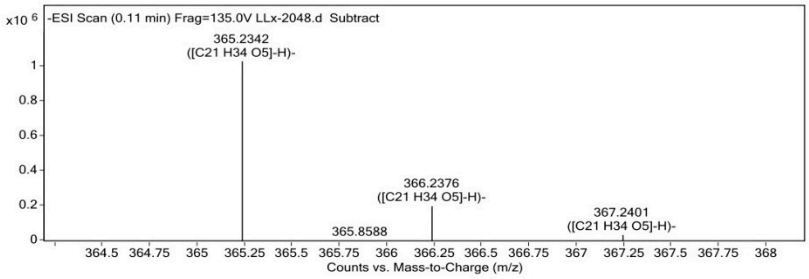

Peak List

m/z	z	Abund	Formula	Ion
365.2342	1	1031285.75	C21 H34 O5	(M-H)-
366.2376	1	200383.92	C21 H34 O5	(M-H)-
367.2401	1	33330.71	C21 H34 O5	(M-H)-
428.2292	1	65403.84		
479.2273	1	211293.08		
480.2302	1	59170.02		
731.4755	1	214296.23		
732.4782	1	99389.41		
733.4798	1	29993.55		
1033.9885	1	64137.72		

Formula Calculator Element Limits

Element	Min	Max
C	3	50
H	0	100
O	0	20

Formula Calculator Results

Formula	CalculatedMass	CalculatedMz	Mz	Diff. (mDa)	Diff. (ppm)	DBE
C21 H34 O5	366.2406	365.2333	365.2342	-0.90	-2.46	5.0000

--- End Of Report ---

Figure S8. High resolution mass spectrum of GA-C14

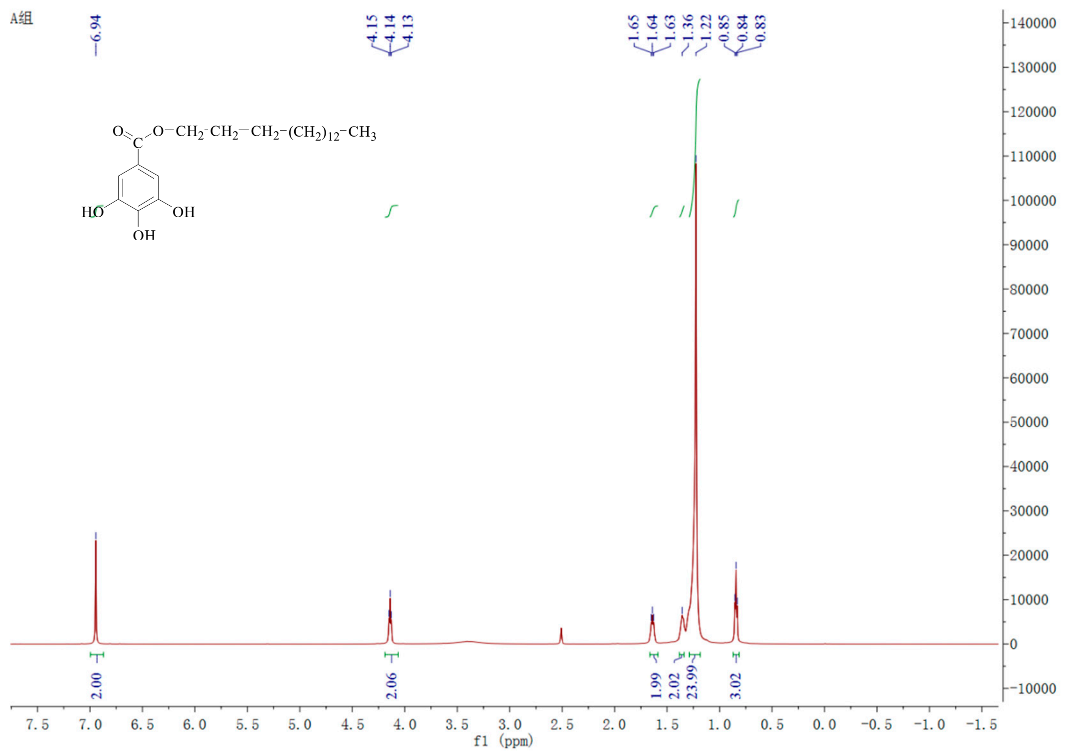

Figure S9. **^{1}H NMR spectroscopy of GA-C16**

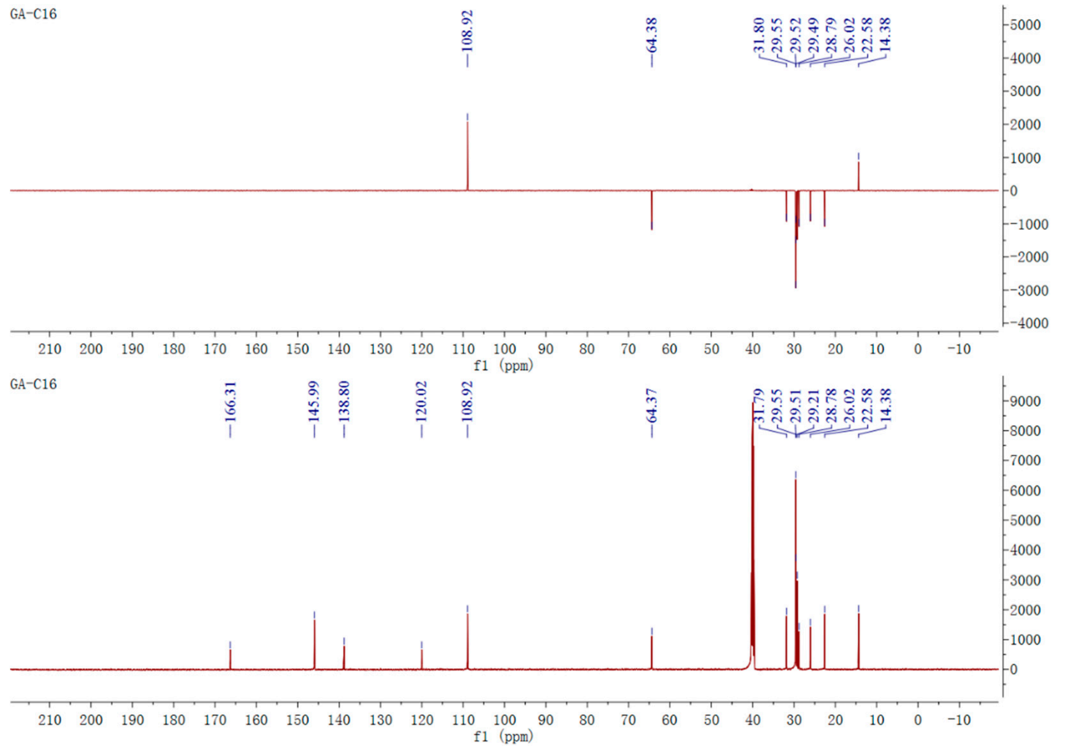

Figure S10. **^{13}C NMR and DEPT spectroscopy of GA-C16**

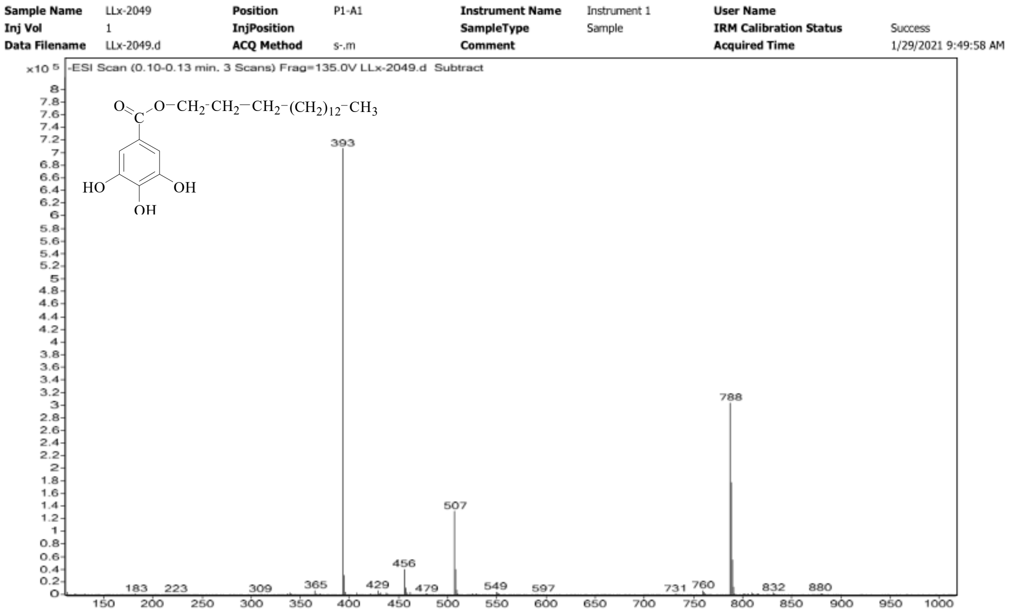

Figure S11. Mass spectrometr of GA-C16

Qualitative Analysis Report

**Data Filename**	LLx-2049.d	**Sample Name**	LLx-2049
**Sample Type**	Sample	**Position**	P1-A1
**Instrument Name**	Instrument 1	**User Name**	
**Acq Method**	s-.m	**Acquired Time**	1/29/2021 9:49:58 AM
**IRM Calibration Status**	Success	**DA Method**	PCDL.m
**Comment**			

Sample Group **Info.**

Acquisition SW 6200 series TOF/6500 series
Version Q-TOF B.05.01 (B5125.2)

User Spectra

Fragmentor Voltage	Collision Energy	Ionization Mode
135	0	ESI

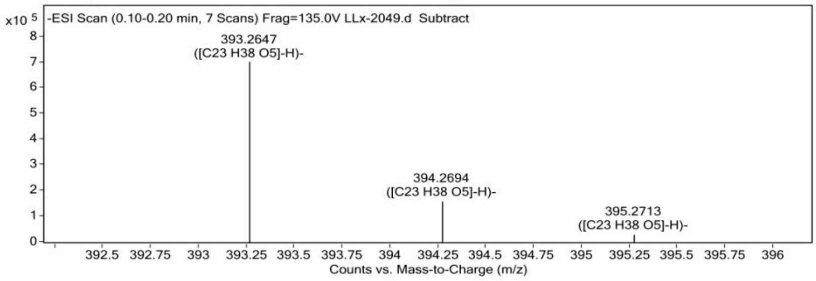

Peak List

m/z	z	Abund	Formula	Ion
393.2647	1	701884.44	C23 H38 O5	(M-H)-
394.2694	1	161749.16	C23 H38 O5	(M-H)-
395.2713	1	32802.64	C23 H38 O5	(M-H)-
456.2607	1	36984.93		
507.2588	1	134905.19		
508.2614	1	42353.3		
787.5389	1	334921.34		
788.5417	1	194604.66		
789.5441	1	62588.24		
1033.9885	1	34992.7		

Formula Calculator Element Limits

Element	Min	Max
C	3	50
H	0	100
O	0	20

Formula Calculator Results

Formula	CalculatedMass	CalculatedMz	Mz	Diff. (mDa)	Diff. (ppm)	DBE
C23 H38 O5	394.2719	393.2646	393.2647	-0.10	-0.25	5.0000

--- End Of Report ---

Figure S12. High resolution mass spectrum of GA-C16

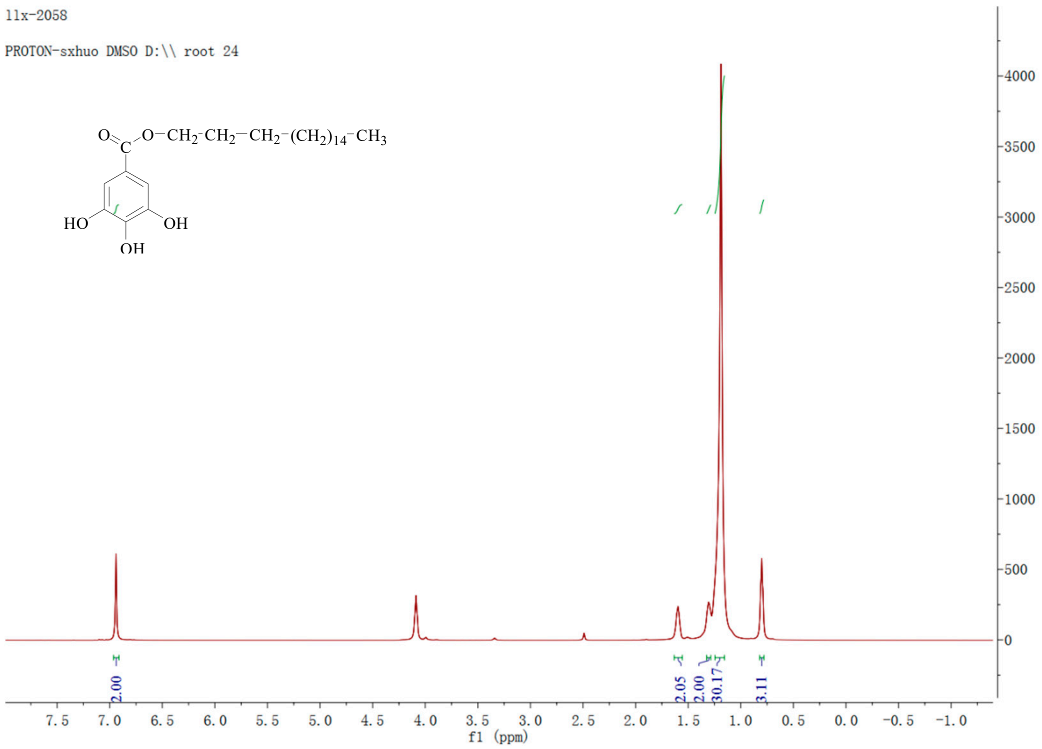

Figure S13. ^{1}H NMR spectroscopy of GA-C18

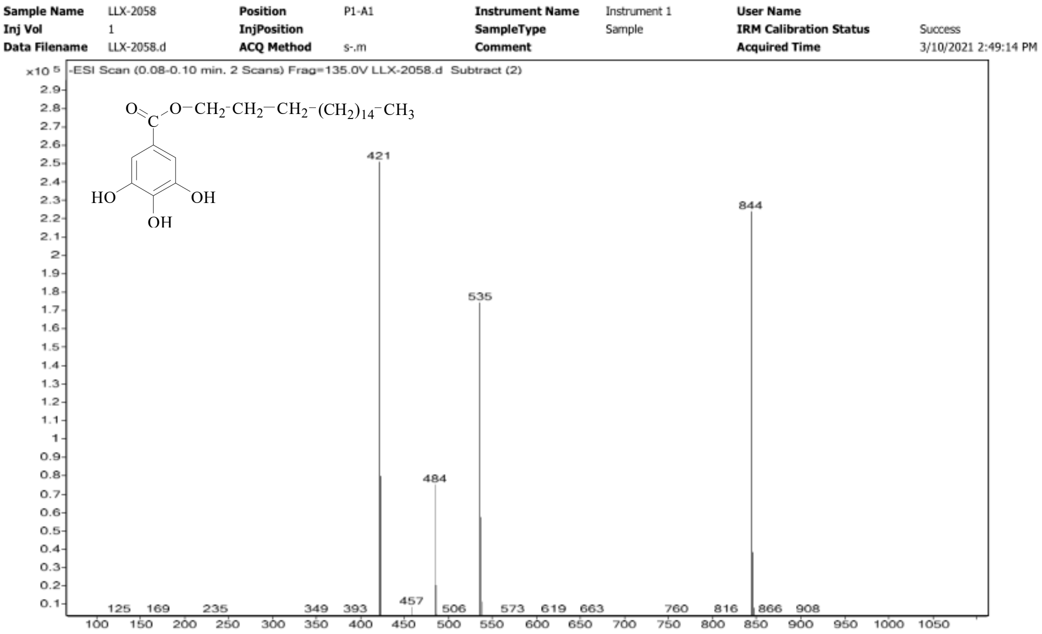

Figure S14. Mass spectrum of GA-C18

Qualitative Analysis Report

**Data Filename**	LLX-2058.d	**Sample Name**	LLX-2058
**Sample Type**	Sample	**Position**	P1-A1
**Instrument Name**	Instrument 1	**User Name**	
**Acq Method**	s-.m	**Acquired Time**	3/10/2021 2:49:14 PM
**IRM Calibration Status**	Success	**DA Method**	PCDL.m
**Comment**			

**Sample Group**		**Info.**
**Acquisition SW Version**	6200 series TOF/6500 series Q-TOF B.05.01 (B5125.2)	

User Spectra

Fragmentor Voltage	Collision Energy	Ionization Mode
135	0	ESI

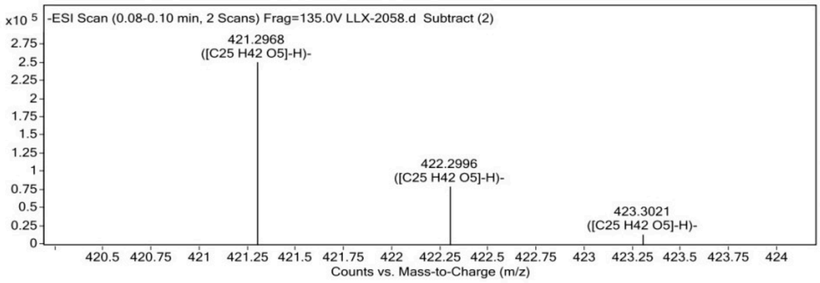

Peak List

m/z	z	Abund	Formula	Ion
421.2968	1	250946.88	C25 H42 O5	(M-H)-
422.2996	1	79717.19	C25 H42 O5	(M-H)-
423.3021	1	14051.7	C25 H42 O5	(M-H)-
484.2916	1	75256.27		
485.2945	1	20465.74		
535.2893	1	174204.19		
536.2923	1	56911.03		
843.5991	1	223634.42		
844.6021	1	127127.66		
845.6047	1	38482.09		

Formula Calculator Element Limits

Element	Min	Max
C	3	50
H	0	100
O	0	20

Formula Calculator Results

Formula	CalculatedMass	CalculatedMz	Mz	Diff. (mDa)	Diff. (ppm)	DBE
C25 H42 O5	422.3032	421.2959	421.2968	-0.90	-2.14	5.0000

--- End Of Report ---

Figure S15. High resolution mass spectrum of GA-C18

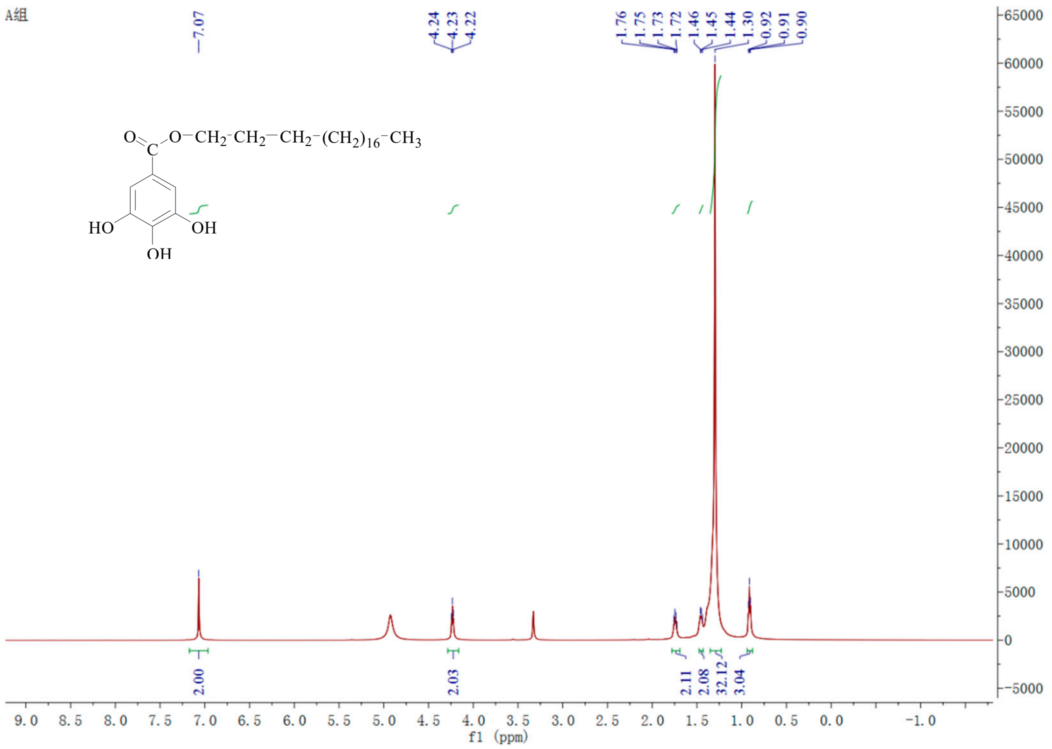

Figure S16. ^{1}H NMR spectroscopy of GA-C20

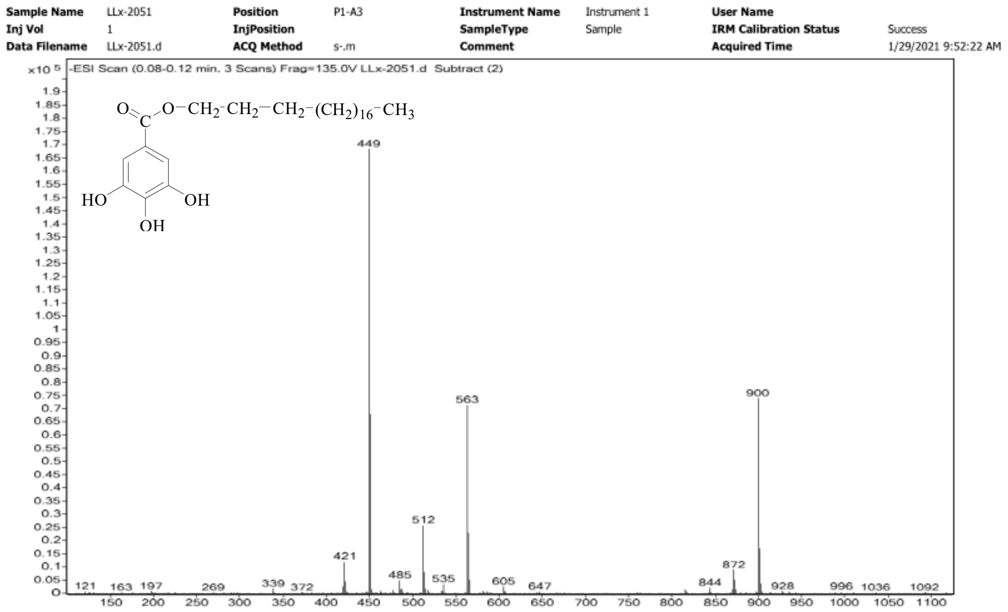

Figure S17. Mass spectrum of GA-C20

Qualitative Analysis Report

**Data Filename**	LLx-2051.d	**Sample Name**	LLx-2051
**Sample Type**	Sample	**Position**	P1-A3
**Instrument Name**	Instrument 1	**User Name**	
**Acq Method**	s-.m	**Acquired Time**	1/29/2021 9:52:22 AM
**IRM Calibration Status**	Success	**DA Method**	PCDL.m
**Comment**			

Sample Group **Info.**

Acquisition SW Version 6200 series TOF/6500 series Q-TOF B.05.01 (B5125.2)

User Spectra

Fragmentor Voltage	Collision Energy	Ionization Mode
135	0	ESI

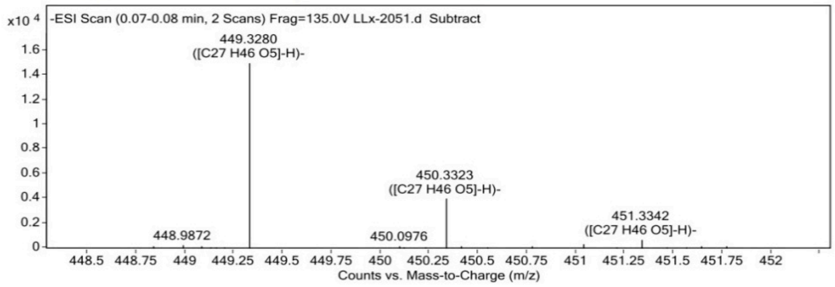

Peak List

m/z	z	Abund	Formula	Ion
119.0359	1	1076.05		
449.328	1	14956.18	C27 H46 O5	(M-H)-
450.3323	1	3996.51	C27 H46 O5	(M-H)-
512.3223	1	3176.09		
513.327	1	902.78		
563.3195	1	4607.83		
564.3171	1	1040.42		
805.9838	1	1810.79		
899.6607	1	1138.85		
1035.9911	1	1039		

Formula Calculator Element Limits

Element	Min	Max
C	3	50
H	0	100
O	0	20

Formula Calculator Results

Formula	CalculatedMass	CalculatedMz	Mz	Diff. (mDa)	Diff. (ppm)	DBE
C27 H46 O5	450.3345	449.3272	449.3280	-0.80	-1.78	5.0000

--- End Of Report ---

Figure S18. High resolution mass spectrum of GA-C20

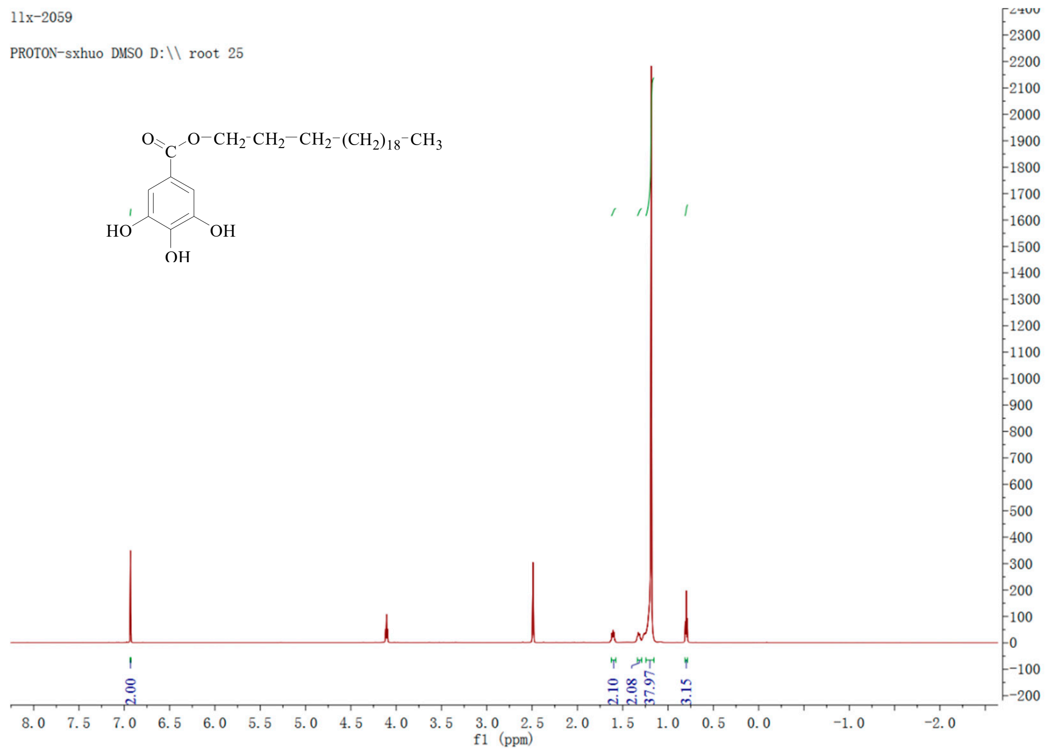

Figure S19. ^{1}H **NMR spectroscopy of GA-C22**

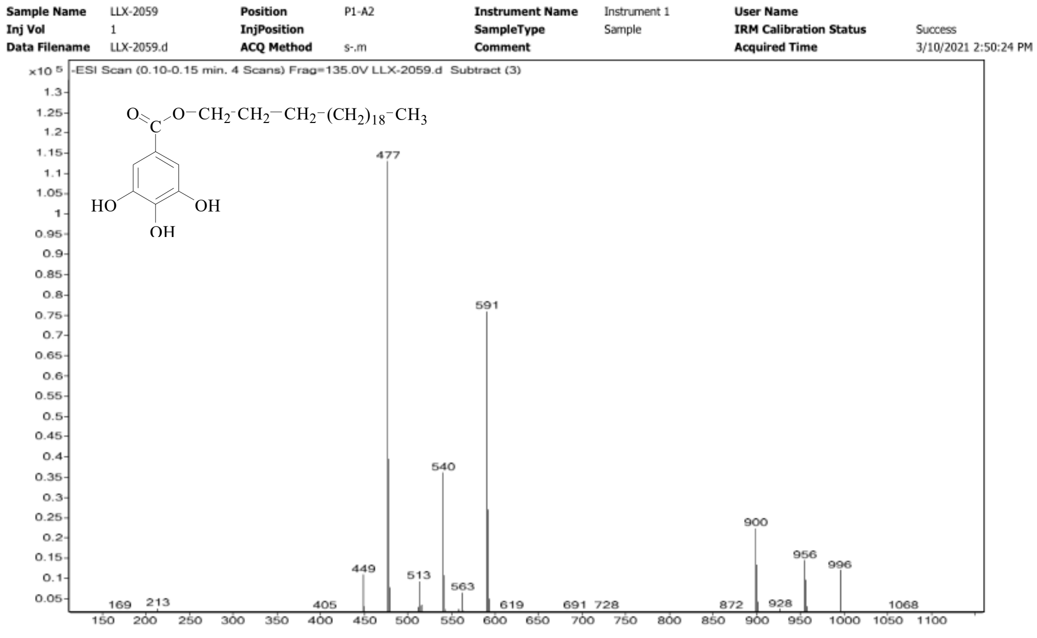

Figure S20. Mass spectrum of GA-C22

Qualitative Analysis Report

**Data Filename**	LLX-2059.d	**Sample Name**	LLX-2059
**Sample Type**	Sample	**Position**	P1-A2
**Instrument Name**	Instrument 1	**User Name**	
**Acq Method**	s-.m	**Acquired Time**	3/10/2021 2:50:24 PM
**IRM Calibration Status**	Success	**DA Method**	PCDL.m
**Comment**			

Sample Group **Info.**

Acquisition SW Version 6200 series TOF/6500 series Q-TOF B.05.01 (B5125.2)

User Spectra

**Fragmentor Voltage**	**Collision Energy**	**Ionization Mode**
135	0	ESI

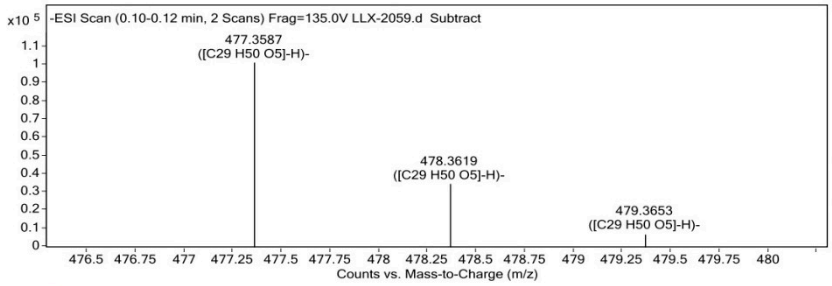

Peak List

m/z	z	Abund	Formula	Ion
421.2962	1	34101.65		
477.3587	1	101149.08	C29 H50 O5	(M-H)-
478.3619	1	34412.46	C29 H50 O5	(M-H)-
535.2888	1	24394.94		
540.3543	1	34074.58		
591.3514	1	68722.31		
592.3545	1	25755.72		
899.6609	1	17960.87		
955.7231	1	12637.76		
1033.9887	1	100475.22		

Formula Calculator Element Limits

Element	Min	Max
C	3	50
H	0	100
O	0	20

Formula Calculator Results

Formula	CalculatedMass	CalculatedMz	Mz	Diff. (mDa)	Diff. (ppm)	DBE
C29 H50 O5	478.3658	477.3585	477.3587	-0.20	-0.42	5.0000

--- End Of Report ---

Figure S21. High resolution mass spectrum of GA-C22

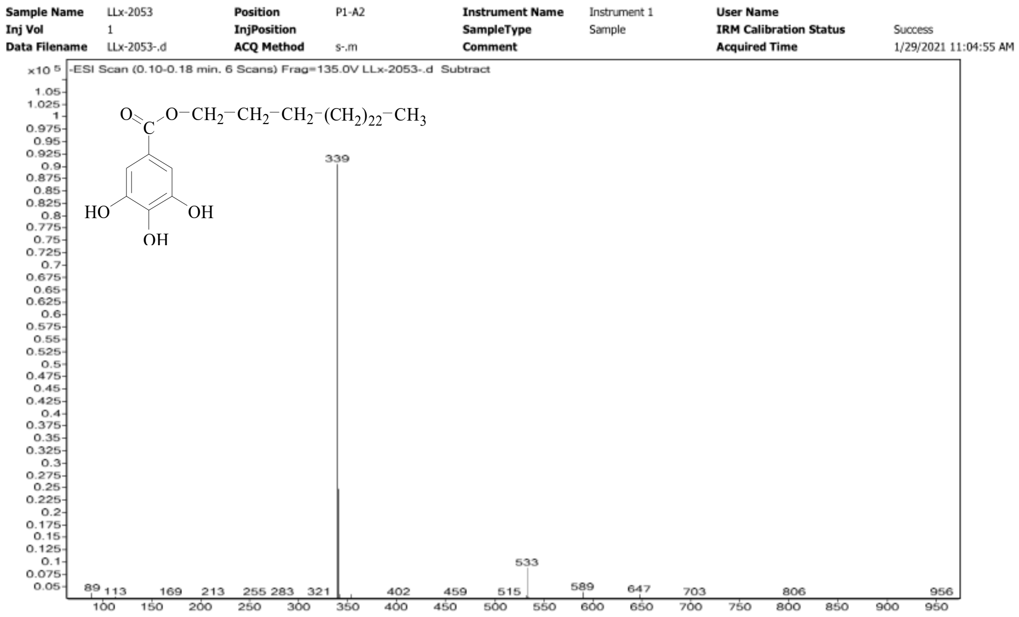

Figure S22. Mass spectrum of GA-C26

Qualitative Analysis Report

**Data Filename**	LLx-2053-.d	**Sample Name**	LLx-2053
**Sample Type**	Sample	**Position**	P1-A2
**Instrument Name**	Instrument 1	**User Name**	
**Acq Method**	s-.m	**Acquired Time**	1/29/2021 11:04:55 AM
**IRM Calibration Status**	Success	**DA Method**	PCDL.m
**Comment**			

Sample Group **Info.**
Acquisition SW Version 6200 series TOF/6500 series Q-TOF B.05.01 (B5125.2)

User Spectra

Fragmentor Voltage	Collision Energy	Ionization Mode
135	0	ESI

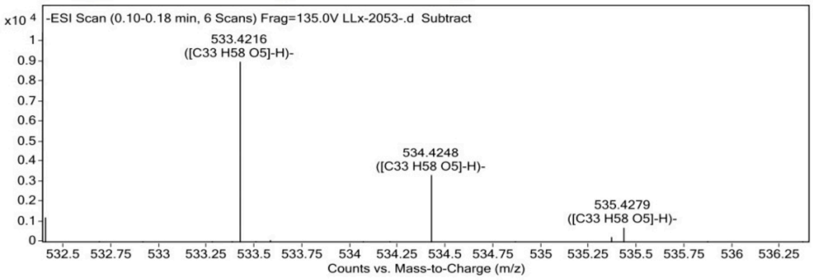

Peak List

m/z	z	Abund	Formula	Ion
89.0244	1	4022.73		
112.9855	1	7139.84		
339.2336	1	92261.14		
340.2367	1	24922.85		
353.2122	1	3373.24		
533.4216	1	8977.72	C33 H58 O5	(M-H)-
589.4831	1	4325.58		
647.4139	1	3591.11		
996.0098	1	12942.43		
1033.9883	1	43494.06		

Formula Calculator Element Limits

Element	Min	Max
C	3	50
H	0	100
O	0	20

Formula Calculator Results

Formula	CalculatedMass	CalculatedMz	Mz	Diff. (mDa)	Diff. (ppm)	DBE
C33 H58 O5	534.4284	533.4211	533.4216	-0.50	-0.94	5.0000

--- End Of Report ---

Figure S23. High resolution mass spectrum of GA-C26

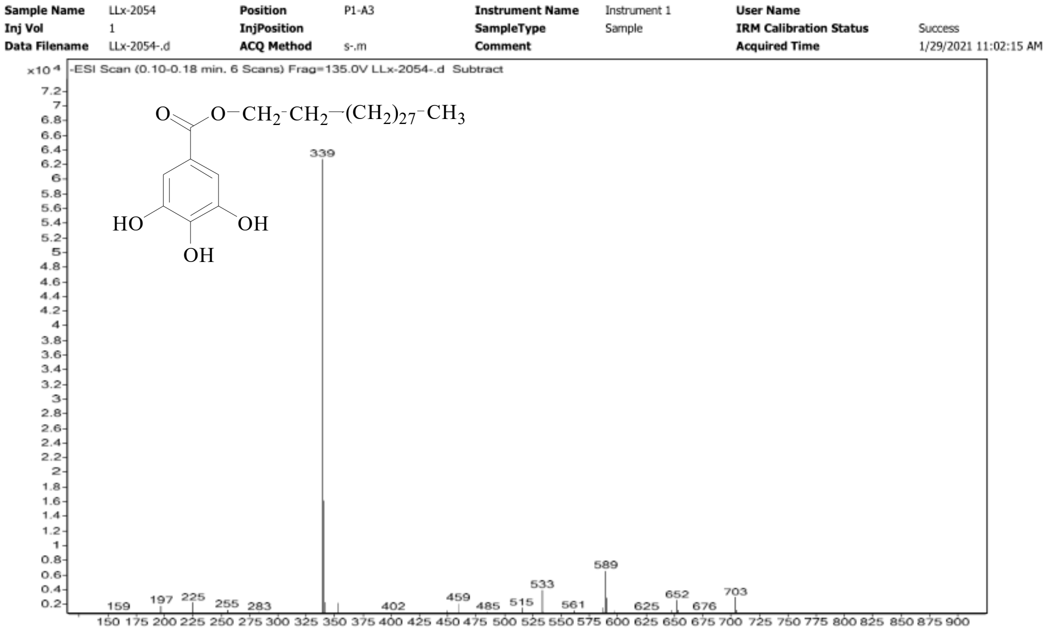

Figure S24. Mass spectrum of GA-C30

Qualitative Analysis Report

**Data Filename**	LLx-2054-.d	**Sample Name**	LLx-2054
**Sample Type**	Sample	**Position**	P1-A3
**Instrument Name**	Instrument 1	**User Name**	
**Acq Method**	s-.m	**Acquired Time**	1/29/2021 11:02:15 AM
**IRM Calibration Status**	Success	**DA Method**	PCDL.m
**Comment**			

Sample Group **Info.**
Acquisition SW 6200 series TOF/6500 series
Version Q-TOF B.05.01 (B5125.2)

User Spectra

**Fragmentor Voltage**	**Collision Energy**	**Ionization Mode**
135	0	ESI

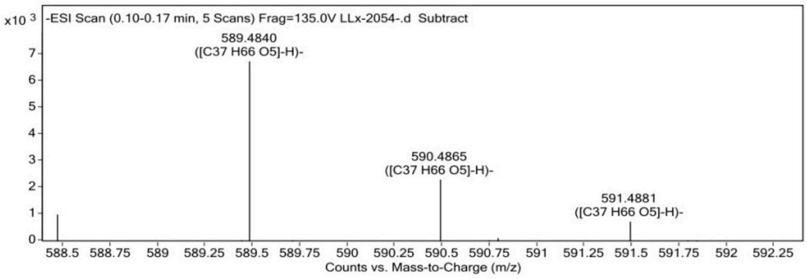

Peak List

m/z	z	Abund	Formula	Ion
112.9855	1	7497.02		
339.2333	1	66748.5		
340.2366	1	17131.37		
533.4209	1	4558.36		
589.484	1	6741.34	C37 H66 O5	(M-H)-
703.4756	1	2901		
996.0099	1	12551.55		
997.0131	1	3196.78		
1033.9882	1	24929.31		
1034.9903	1	3629.87		

Formula Calculator Element Limits

Element	Min	Max
C	3	50
H	0	100
O	0	20

Formula Calculator Results

Formula	CalculatedMass	CalculatedMz	Mz	Diff. (mDa)	Diff. (ppm)	DBE
C37 H66 O5	590.4910	589.4837	589.4840	-0.30	-0.51	5.0000

--- End Of Report ---

Figure S25. High resolution mass spectrum of GA-C30

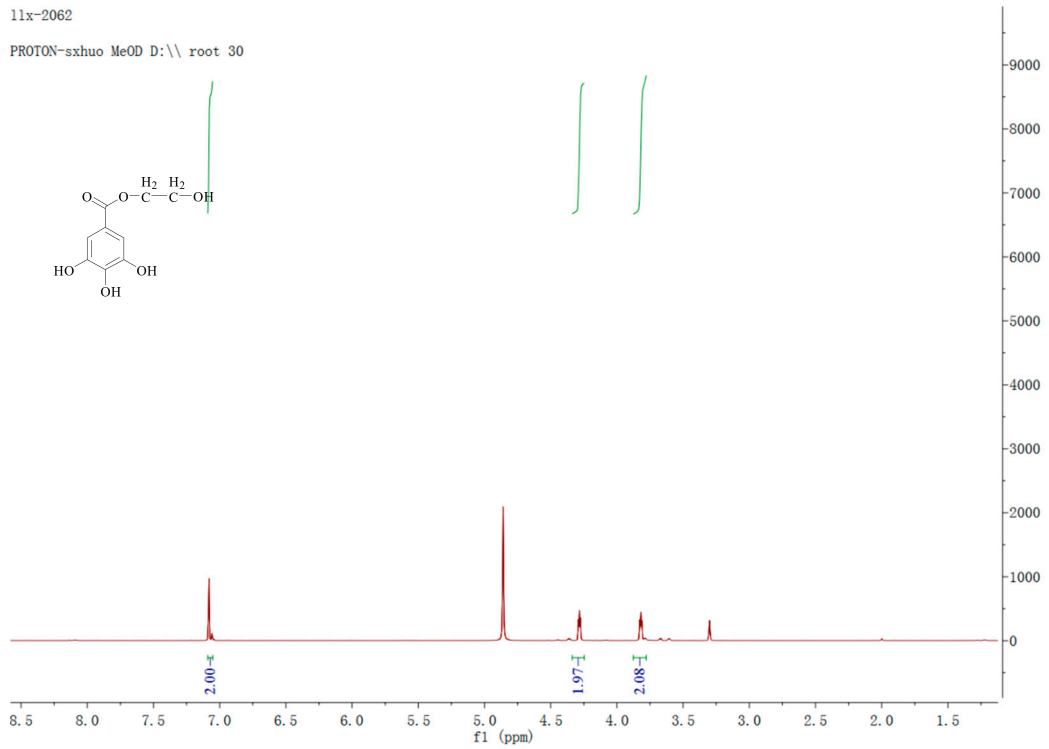

Figure S26. [1]**H NMR spectroscopy of GA-EG**

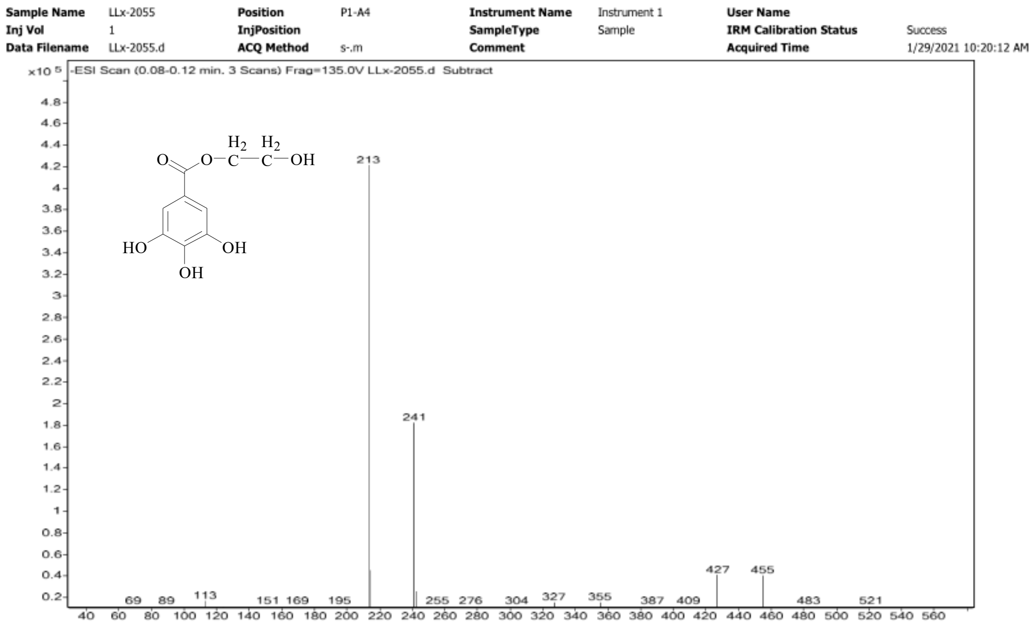

Figure S27. Mass spectrum of GA-EG

Qualitative Analysis Report

**Data Filename**	LLx-2055.d	**Sample Name**	LLx-2055
**Sample Type**	Sample	**Position**	P1-A4
**Instrument Name**	Instrument 1	**User Name**	
**Acq Method**	s-.m	**Acquired Time**	1/29/2021 10:20:12 AM
**IRM Calibration Status**	Success	**DA Method**	PCDL.m
**Comment**			

Sample Group **Info.**

Acquisition SW Version 6200 series TOF/6500 series Q-TOF B.05.01 (B5125.2)

User Spectra

Fragmentor Voltage	Collision Energy	Ionization Mode
135	0	ESI

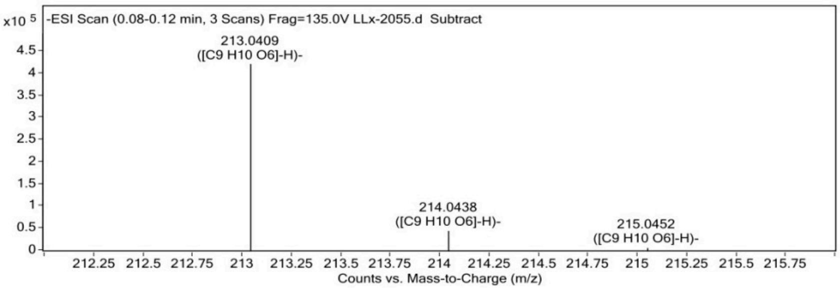

Peak List

m/z	z	Abund	Formula	Ion
112.9855	1	16503.08		
213.0409	1	421246	C9 H10 O6	(M-H)-
214.0438	1	45389.48	C9 H10 O6	(M-H)-
241.036	1	182265.61		
242.0386	1	25904.52		
327.0331	1	14933.36		
355.0281	1	14969.18		
427.0882	1	40691.2		
455.0834	1	40059		
1033.9884	1	17082.92		

Formula Calculator Element Limits

Element	Min	Max
C	3	50
H	0	100
O	0	20

Formula Calculator Results

Formula	CalculatedMass	CalculatedMz	Mz	Diff. (mDa)	Diff. (ppm)	DBE
C9 H10 O6	214.0477	213.0405	213.0409	-0.40	-1.88	5.0000

--- End Of Report ---

Figure S28. High resolution mass spectrum of GA-EG

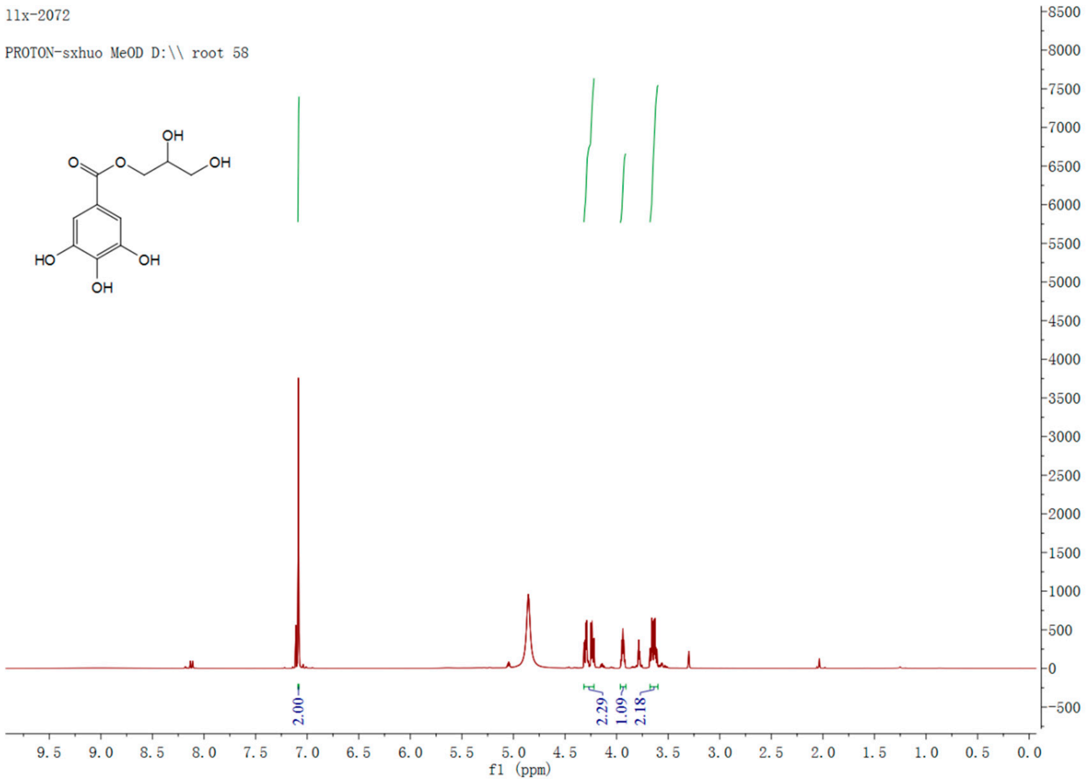

Figure S29. ¹H NMR spectroscopy of GA-GL

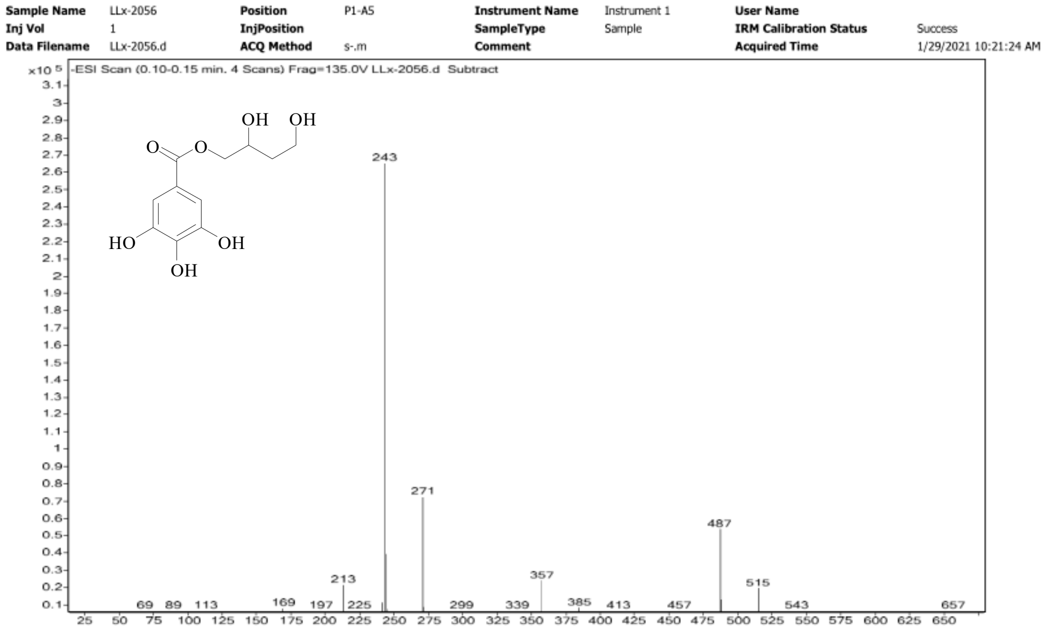

Figure S30. Mass spectrum of GA-GL

Qualitative Analysis Report

**Data Filename**	LLx-2056.d	**Sample Name**	LLx-2056
**Sample Type**	Sample	**Position**	P1-A5
**Instrument Name**	Instrument 1	**User Name**	
**Acq Method**	s-.m	**Acquired Time**	1/29/2021 10:21:24 AM
**IRM Calibration Status**	Success	**DA Method**	PCDL.m
**Comment**			

Sample Group **Info.**
Acquisition SW 6200 series TOF/6500 series
Version Q-TOF B.05.01 (B5125.2)

User Spectra

Fragmentor Voltage	Collision Energy	Ionization Mode
135	0	ESI

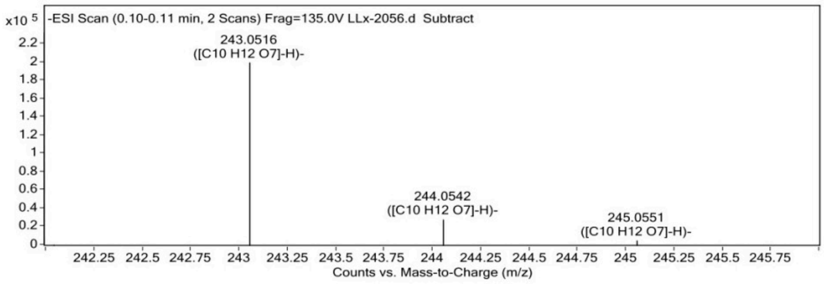

Peak List

m/z	z	Abund	Formula	Ion
213.0399	1	15019.34		
241.035	1	8374.93		
243.0516	1	199772.34	C10 H12 O7	(M-H)-
244.0542	1	28526.13	C10 H12 O7	(M-H)-
271.0461	1	52460.76		
272.0489	1	7252.55		
357.0438	1	18324.95		
487.1091	1	34451.7		
488.112	1	7455.41		
515.1044	1	12105.68		

Formula Calculator Element Limits

Element	Min	Max
C	3	50
H	0	100
O	0	20

Formula Calculator Results

Formula	CalculatedMass	CalculatedMz	Mz	Diff. (mDa)	Diff. (ppm)	DBE
C10 H12 O7	244.0583	243.0510	243.0516	-0.60	-2.47	5.0000

--- End Of Report ---

Figure S31. High resolution mass spectrum of GA-GL

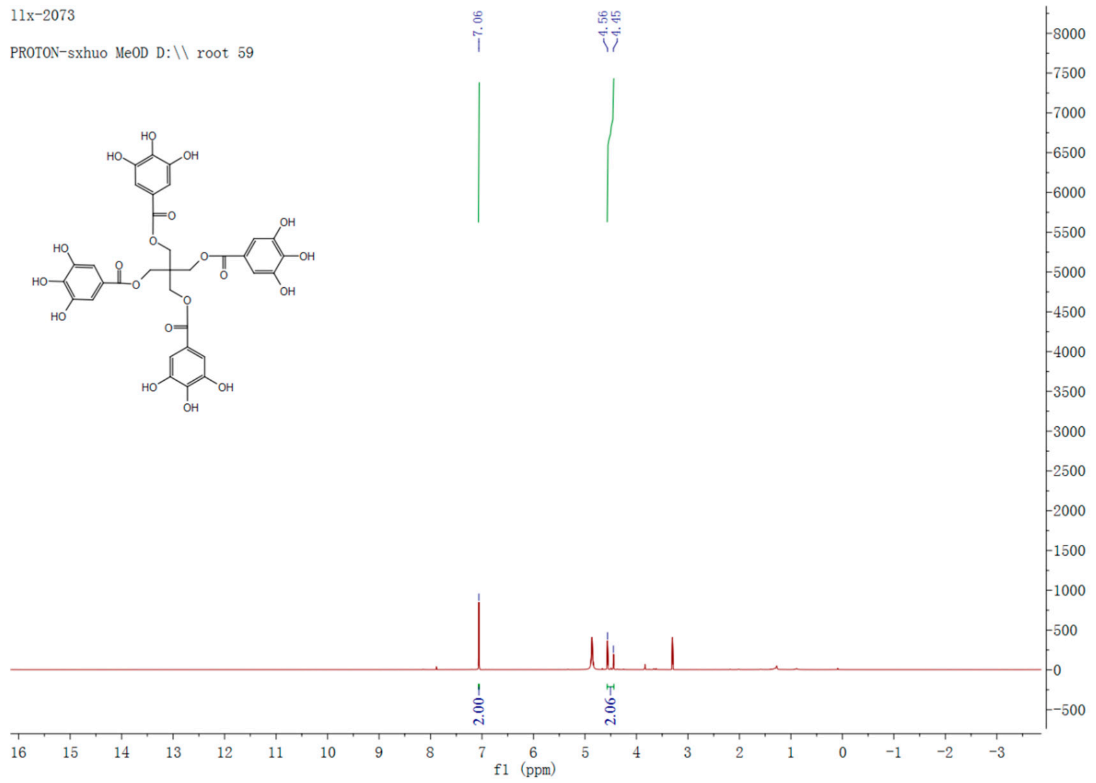

Figure S32. [1]**H NMR spectroscopy of GA-PT**

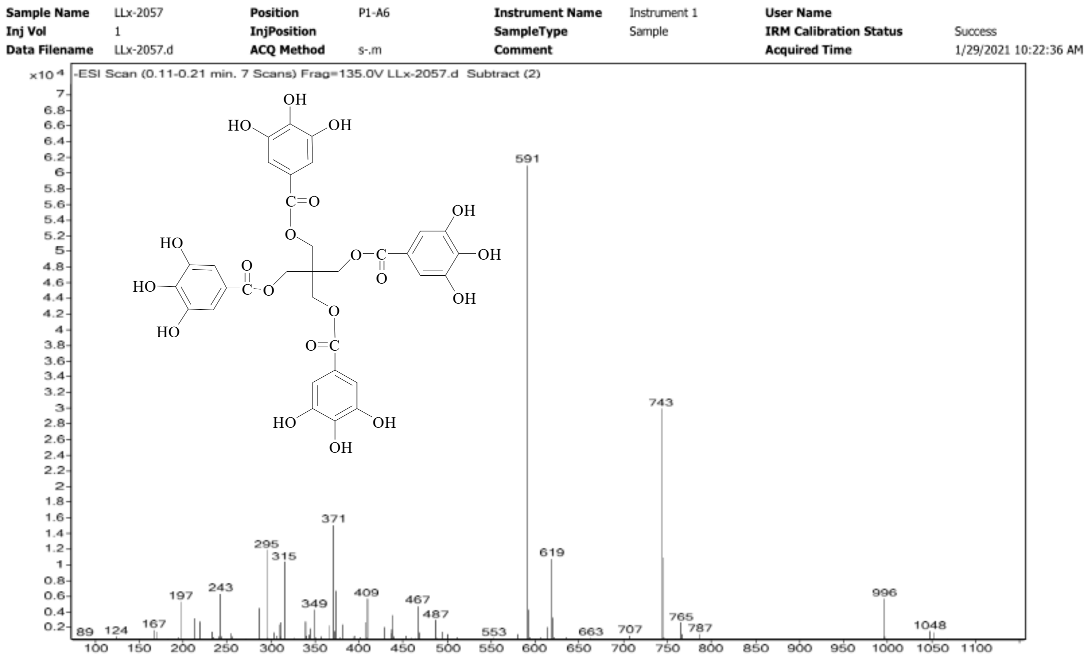

Figure S33. Mass spectrum of GA-PT

Qualitative Analysis Report

**Data Filename**	LLx-2057.d	**Sample Name**	LLx-2057
**Sample Type**	Sample	**Position**	P1-A6
**Instrument Name**	Instrument 1	**User Name**	
**Acq Method**	s-.m	**Acquired Time**	1/29/2021 10:22:36 AM
**IRM Calibration Status**	Success	**DA Method**	PCDL.m
**Comment**			

Sample Group **Info.**

Acquisition SW Version 6200 series TOF/6500 series Q-TOF B.05.01 (B5125.2)

User Spectra

Fragmentor Voltage	Collision Energy	Ionization Mode
135	0	ESI

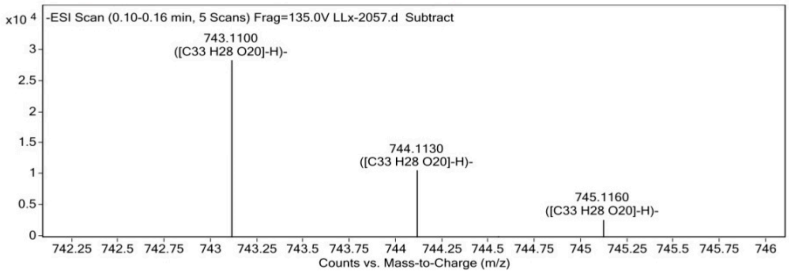

Peak List

m/z	z	Abund	Formula	Ion
295.0464	1	11377.45		
315.0724	1	10497.89		
371.0528	1	13836.7		
373.114	1	6795.98		
591.0993	1	59426.69		
592.1023	1	18911		
619.0941	1	10645.51		
743.11	1	28349.77	C33 H28 O20	(M-H)-
744.113	1	10615.76	C33 H28 O20	(M-H)-
1033.9883	1	21648.38		

Formula Calculator Element Limits

Element	Min	Max
C	3	50
H	0	100
O	0	20

Formula Calculator Results

Formula	CalculatedMass	CalculatedMz	Mz	Diff. (mDa)	Diff. (ppm)	DBE
C33 H28 O20	744.1174	743.1101	743.1100	0.10	0.13	20.0000

--- End Of Report ---

Figure S34. High resolution mass spectrum of GA-PT
